# A convenient enantioselective decarboxylative aldol reaction to access chiral α-hydroxy esters using β-keto acids

**DOI:** 10.3762/bjoc.10.95

**Published:** 2014-04-29

**Authors:** Zhiqiang Duan, Jianlin Han, Ping Qian, Zirui Zhang, Yi Wang, Yi Pan

**Affiliations:** 1 School of Chemistry and Chemical Engineering, Nanjing University, Nanjing, 210093, China; 2 Institute for Chemistry & Biomedical Sciences, Nanjing University, Nanjing, 210093, China; 3 State of Key Laboratory of Coordination, Nanjing University, Nanjing, 210093, China

**Keywords:** enantioselective synthesis, hydroxy esters, scandium

## Abstract

We show a convenient decarboxylative aldol process using a scandium catalyst and a PYBOX ligand to generate a series of highly functionalized chiral α-hydroxy esters. The protocol tolerates a broad range of β-keto acids with inactivated aromatic and aliphatic α-keto esters. The possible mechanism is rationalized.

## Introduction

**Figure 1:**
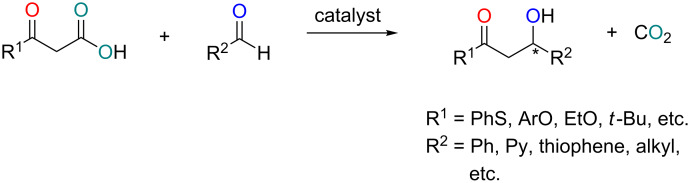
Decarboxylative aldol reactions of β-keto acids with aldehydes.

The catalytic enantioselective construction of tertiary carbon centres is a major challenge in organic chemistry. The nucleophilic attack of carbonyls appears as a common procedure, affording chiral tertiary alcohols which are ubiquitous in the biological sciences and pharmaceutical industry [1–6]. The decarboxylative aldol reaction, broadly used for the generation of ester enolate equivalents by the promotion of releasing CO_2_, has become an appealing method to access chiral tertiary alcohols. Taking advantage of this rigid reactivity, several unique catalytic decarboxylative aldol transformations of β-keto acids with various protic aldehydes have been developed [7–10] (Figure 1). High enantioselectivities were achieved with one point-binding aldehydes and two-point binding β-keto acids under mild reaction conditions. The lack of strong Lewis acids or very basic intermediates enabled it to tolerate functionalities that would normally be incompatible with ester enolates, for instance, hydroxy groups, phenols, enolizable aldehydes and carboxylic acids.

Other less reactive carbonyl derivatives such as isatins [11–12], ketimines [13] and sulfonylimines [14] have also been employed with β-keto acids in the decarboxylative addition processes.

**Figure 2:**
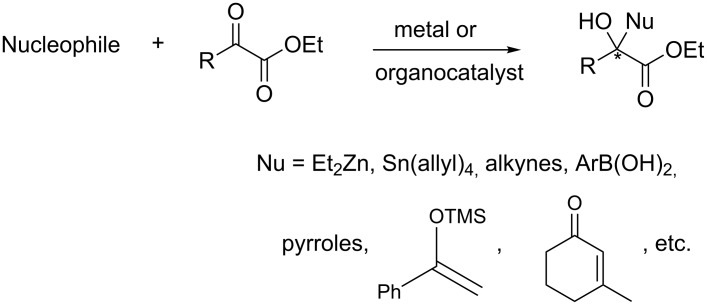
Nucleophilic reaction of α-keto esters to generate tertiary alcohols.

α-Keto esters as surrogates of aldehydes for the generation of chiral alcohols by stereocontrolled nucleophilic alkylation [15–19], alkynylation [20–21], 1,2-addition [22–26] and aldol reaction [27–32] have been developed. Various nucleophiles such as organometallics, boronic acids and unsaturated ketones can be tolerated in this context (Figure 2).

**Figure 3:**
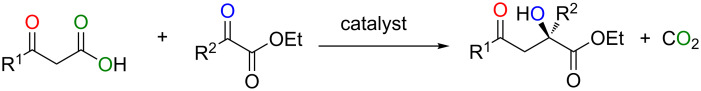
Decarboxylative aldol reactions of β-keto acids with α-keto esters.

We presume that relatively hindered α-keto esters could also be engaged as aldehydes in the decarboxylative aldol reactions with β-keto acids, which would provide a practical and efficient route to access α-hydroxy esters in an enantioseletive fashion (Figure 3).

## Results and Discussion

By investigating different Lewis acids with various chiral PyBox ligands **4**–**8** (Table 1), we discovered that Sc(OTf)_3_ and tridentate PyBox ligand **6a** could promote the decarboxylative aldol reaction of β-keto acid **1a** with α-keto ester **2a** in excellent yield with high enantioselectivity in toluene (Table 1, entry 9). Trace amount of side product acetophenone was formed through decarboxylative protonation of β-keto acid **1a**, which was commonly observed in the case of chiral organic base catalysed decarboxylative additions.

**Table 1:** Evaluation of ligands and optimisation of reaction conditions.^a^

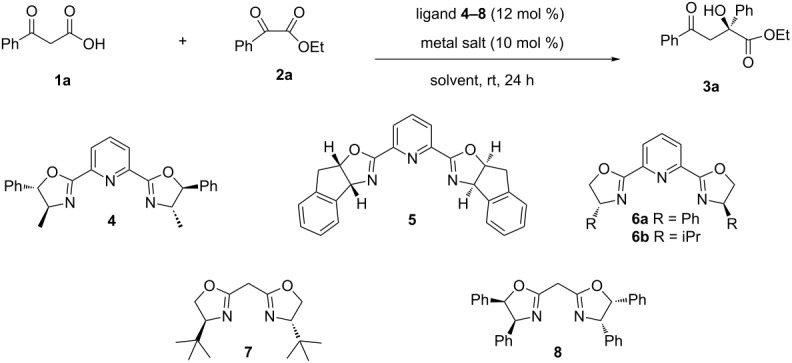					

Entry	Ligand	Metal salt	Solvent	Yield (%)^b^	ee (%)^c^

1	**4**	Sc(OTf)_3_	toluene	93	27
2	**4**	Yb(OTf)_3_	toluene	90	19
3	**4**	La(OTf)_3_	toluene	90	11
4	**4**	In(OTf)_3_	toluene	88	5
5	**4**	Hf(OTf)_4_	toluene	91	15
6	**7**	Cu(OTf)_2_	toluene	85	5
7	**8**	Cu(OTf)_2_	toluene	83	17
8	**5**	Sc(OTf)_3_	toluene	91	33
9	**6a**	Sc(OTf)_3_	toluene	95	76
10	**6b**	Sc(OTf)_3_	toluene	94	45
11	**6a**	Sc(OTf)_3_	CH_2_Cl_2_	90	49
12	**6a**	Sc(OTf)_3_	CHCl_3_	93	79
13	**6a**	Sc(OTf)_3_	CH_3_CN	88	33
14	**6a**	Sc(OTf)_3_	THF	89	27
15	**6a**	Sc(OTf)_3_	CHCl_3_	91	62^d^
16	**6a**	Sc(OTf)_3_	CHCl_3_	95	84^e^

^a^Reaction conditions: **1a** (0.2 mmol), **2a** (0.1 mmol), metal salt (10 mol %), ligand (12 mol %). ^b^Isolated yield after column chromatography. ^c^Determined by HPLC analysis using a chiralcel IA column. ^d^10 mg 4 Å molecular sieves were added. ^e^At 0 °C for 48 h.

Further optimisation of the reaction conditions showed that CHCl_3_ was the best solvent choice in terms of catalytic activity and asymmetric induction (Table 1, entry 12). Lowering the reaction temperature from 20 °C to 0 °C increased the ee value from 79% to 84% (Table 1, entry 16). The addition of 4 Å molecular sieves was not able to accelerate the reaction or to improve the enantioselectivity (Table 1, entry 15).

**Scheme 1:**
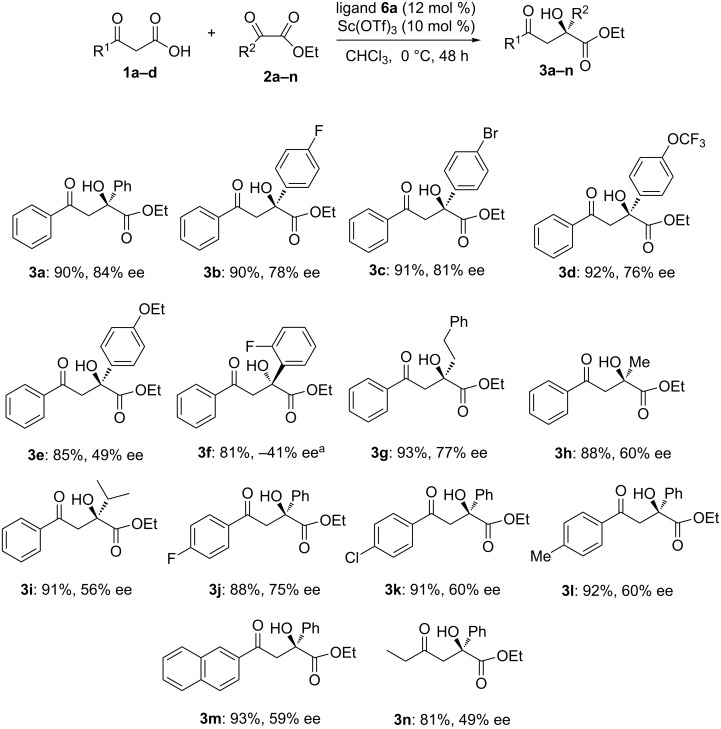
Asymmetric decarboxylative aldol reaction of various β-keto acids with α-keto esters under optimised conditions. Reaction conditions: **1** (0.2 mmol), **2** (0.1 mmol), Sc(OTf)_3_ (10 mol %), ligand **6a** (12 mol %). Isolated yield after column chromatography. Enatiomeric excess determined by HPLC analysis using a chiralcel column. ^a^4(*S*)-PyBox **6a** was used.

The reaction scope was investigated by using different aryl and alkyl substituted β-keto acids and α-keto esters. Evaluating the results of products **3b–f**, suggested that the α-keto esters with electron-withdrawing substituents were more favoured than those with electron-donating groups (Scheme 1, **3b–e**). The ortho substituted phenyl α-keto ester gave a lower ee (41%) than those with para substituents (49–84% ee, **3b–e**). Aliphatic α-keto esters provided the corresponding aldol products with moderate enantioselectivity (56–77% ee, **3g–i**). Also, different β-keto acids **1a–d** with aromatic and alkyl substituents afforded chiral hydoxy esters **3j–n** in high yields and good enantioselectivities (49–75%).

We also examined different R^3^ groups of α-keto esters **2a–d**. Under the established conditions, an ethyl group afforded the highest yield with the best selectivity. The enantioselectivity did not improve in the cases of methyl, isopropyl or benzyl esters (Table 2, entries 2–4).

**Table 2:** Effect of the ester group on the α-keto esters with β-keto acid **1a**.^a^

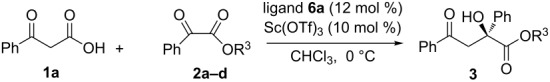				

Entry	R^3^	Time (h)	Yield (%)^b^	ee (%)^c^

1	Et, **2a**	48	95	84, **3a**
2	Me, **2b**	36	93	47, **3o**
3	iPr, **2c**	48	91	71, **3p**
4	Bn, **2d**	48	89	67, **3q**

^a^Reaction conditions: **1a** (0.2 mmol), α-keto esters **2** (0.1 mmol), scandium (10 mol %), and ligand **6a** (12 mol %). ^b^Isolated yield after column chromatography. ^c^Determined by HPLC analysis using chiralcel column.

**Scheme 2:**
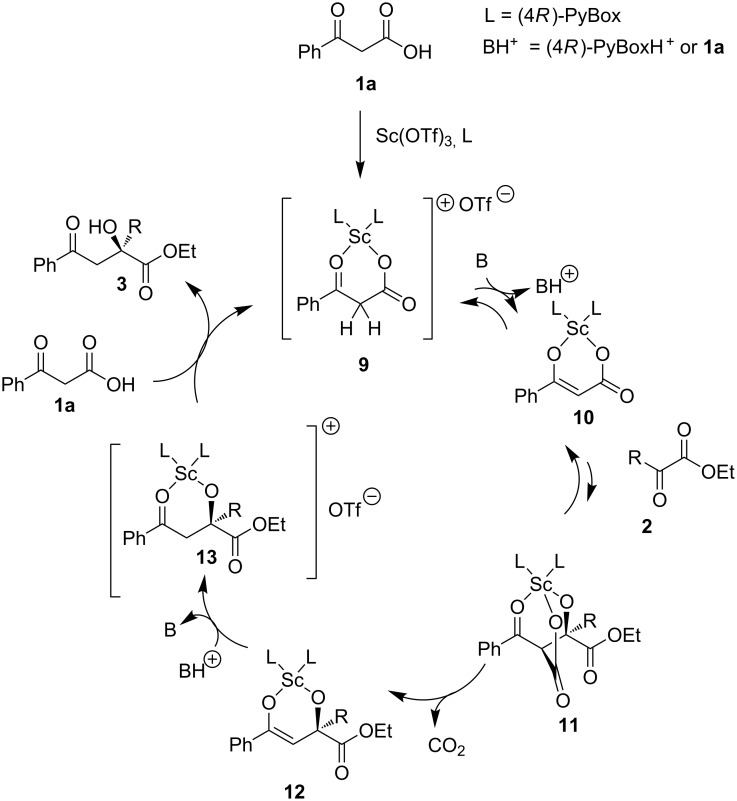
Proposed mechanism of decarboxylative aldol reaction.

The mechanism of the reaction was proposed based on the kinetic studies of the malonic acid half thioester system by Shair [33]. Essentially β-keto acids can undergo decarboxylation or deprotonation to generate enolates. Though in the case of enzymatic reactions, decarboxylation occurs first to form the enolates, followed by condensation with esters; it is believed that in the scandium-catalysed aldol process of β-keto acid, similar to the case of malonic acid half thioesters, decarboxylation happens after the addition to the ester (Scheme 2). First, deprotonation and enolisation of **9** followed by addition of α-keto ester **2** gives intermediate **11**. After decarboxylation to afford **12**, a protonation step occurs late in the reaction pathway to form the aldol product **3** and completes the mechanistic cycle.

## Conclusion

We have described a new convenient decarboxylative aldol protocol to generate highly functionalised chiral α-hydroxy esters employing a Sc(OTf)_3_ and PyBox catalytic system. A broad range of inactivated α-keto esters were proven to be tolerated. The possible mechanism of the reaction was also rationalized. Further investigations to explore the reaction scope are underway.

## Supporting Information

File 1 Experimental and analytical data.
