# Supplementary material for: A convenient enantioselective decarboxylative aldol reaction to access chiral α-hydroxy esters using β-keto acids
Source: Beilstein J Org Chem. 2014 Apr 29;10:969–74. doi: 10.3762/bjoc.10.95 (PMC4077541; doi:10.3762/bjoc.10.95)
Supplement: File 1 — Experimental and analytical data. [file Beilstein_J_Org_Chem-10-969-s001.pdf]

Supporting Information

for

A convenient enantioselective decarboxylative aldol reaction to access chiral α-hydroxy esters using β-keto acids

Zhiqiang Duan[1], Jianlin Han[1,2], Ping Qian[1], Zirui Zhang[1],Yi Wang*[1,3] and Yi Pan[1,3]

Address: [1]School of Chemistry and Chemical Engineering, Nanjing University, Nanjing, 210093, China, [2]Institute for Chemistry & Biomedical Sciences, Nanjing University, Nanjing, 210093, China and [3]State of Key Laboratory of Coordination, Nanjing University, Nanjing, 210093,China

Email: Yi Wang - yiwang@nju.edu.cn

*Corresponding author

Experimental and analytical data

Table of contents

1. General information……………………………………………………….......S2
2. Procedure for the Preparation of α-hydroxyesters………………………….S2
3. ^{1}H and ^{13}C NMR spectra for compound **3a**–**3q**……………………………S13
4. HPLC of compound **3a**–**3q**……………………………………………………..S30

1. General information

Flash chromatography was performed using silica gel 60 (200-300 mesh). Thin layer chromatography was carried out on silica gel 60 F-254 TLC plates of 20 cm × 20 cm. Melting points are uncorrected. IR spectra were collected on Bruker Vector 22 in KBr pellets. Values of optical rotation were measured on Rudolph Automatic Polarimeter A21101 at the wavelength of the sodium D-line (589 nm) at 25 °C. ^{1}H and ^{13}C NMR (TMS used as internal standard) spectra were recorded with a Bruker ARX 400 spectrometer. High resolution mass spectra for all the new compounds were done by Micromass Q-Tof instrument (ESI). HPLC analysis was performed on Shimadzu SPD-20A using Daicel Chiralpak OD-H or IA column.

Organic solvents used were dried by standard methods when necessary. β-keto acids [1-2] and α-keto esters [3] were prepared according to the previous reports.

2. General procedure for the decarboxylative aldol reaction

A dry Schlenk tube under N_2 atmosphere was charged with $Sc(OTf)_3$ (0.01 mmol, 0.1 equiv.) and pyBOX 6a (0.012 mmol, 0.12 equiv.). Dry $CHCl_3$ (0.5 mL) was added and the solution was stirred at 0°C for 30 min. Subsequently, α-keto ester (0.1 mmol), β-keto acid (0.2 mmol, 2.0 equiv.) were added and the Schlenk tube was sealed. After complete consumption of starting material (0°C, 48 hours, TLC control), the solvent was evaporated under reduced pressure, the residue was purified by a flash column chromatography on silica gel to afford the desired adducts and the ee values were determined by HPLC analysis with chiral column.

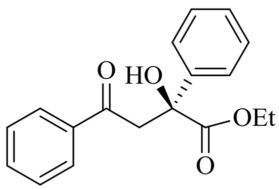

Compound **3a**: colorless solid, yield 90%, 84% ee, mp 67-69 °C, $[\alpha]_D^{25}$ -30.00 (*c* = 0.20, $CHCl_3$). ^1H NMR (400 MHz, $CDCl_3$): δ = 7.97-7.94 (m, 2H), 7.69-7.67 (m, 2H), 7.61-7.57 (m, 1H), 7.48-7.31(m, 5H), 4.51 (s, 1H), 4.26 (q, *J* = 7.1 Hz, 2H), 4.06 (d, *J* = 17.7 Hz, 1H), 3.58 (d, *J* = 17.7 Hz, 1H), 1.25 (t, *J* = 7.1 Hz, 3H). ^{13}C NMR (101 MHz, $CDCl_3$): δ = 198.66, 174.01, 140.72, 136.41, 133.73, 128.71, 128.52, 128.20, 128.08, 125.11, 76.44, 62.24, 49.00, 14.01. IR (KBr): ν = 3492, 3060, 2956, 1723, 1676, 1596, 1448, 1365, 1275, 1209, 1138, 1097, 1066, 1001, 949, 752, 701, 685, 658, 578. HRMS [M+Na$^+$]: calcd for $C_{18}H_{18}O_4Na$: 321.1103, found: 321.1105. HPLC analysis (IA column, λ = 254 nm, eluent: hexane/2-propanol 80/20, flow rate: 0.8 mL/min): t_R = 10.80 min (minor), 17.04 min (major).

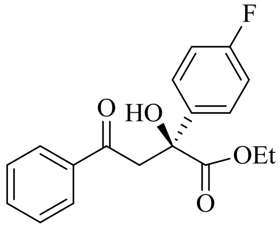

Compound **3b**: colorless solid, yield 90%, 78% ee, mp 90-91 °C, $[\alpha]_D^{25}$ -77.29 (*c* = 0.41, $CHCl_3$). ^1H NMR (400 MHz, $CDCl_3$): δ = 7.95-7.92 (m, 2H), 7.67-7.64 (m, 2H), 7.58-7.54 (m, 1H), 7.45-7.42 (m, 2H), 7.07-7.03 (m, 2H), 4.57 (s, 1H), 4.23 (q, *J* = 7.1 Hz, 2H), 4.04 (d, *J* = 17.7 Hz, 1H), 3.55 (d, *J* = 17.7 Hz, 1H), 1.22 (t, *J* = 7.1 Hz, 3H). ^{13}C NMR (101 MHz, $CDCl_3$): δ = 198.45, 173.87, 163.77, 161.31, 136.58, 136.55, 136.31, 133.80, 128.73, 128.19, 127.14, 127.06, 115.40, 115.19, 76.07, 62.28, 49.00, 13.98. IR (KBr): ν = 3512, 3096, 2923, 1731, 1679, 1597, 1508, 1449, 1353, 1273, 1212, 1141, 1063, 1011, 846, 757, 690, 624, 562. HRMS [M+Na$^+$]: calcd for $C_{18}H_{17}FO_4Na$: 339.1009, found: 339.1004. HPLC analysis (IA column, λ = 254 nm, eluent: hexane/2-propanol 80/20, flow rate: 0.8 mL/min): t_R = 11.06 min (minor), 17.83 min (major).

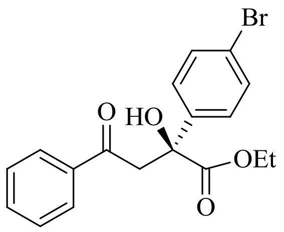

Compound **3c**: colorless oil, yield 91%, 81% ee, $[\alpha]_D^{25}$ -62.53 (c = 0.39, $CHCl_3$). ^1H NMR (400 MHz, $CDCl_3$): δ = 7.93-7.91 (m, 2H), 7.58-7.53 (m, 3H), 7.50-7.40 (m, 4H), 4.58 (s, 1H), 4.22 (q, J = 7.1 Hz, 2H), 4.01 (d, J = 17.7 Hz, 1H), 3.54 (d, J = 17.7 Hz, 1H), 1.21 (t, J = 7.1 Hz, 3H). ^{13}C NMR (101 MHz, $CDCl_3$): δ = 198.28, 173.60, 139.92, 136.25, 133.84, 131.59, 128.75, 128.21, 127.13, 122.28, 76.14, 62.38, 48.85, 14.03. IR (KBr): ν = 3506, 2980, 2934, 1731, 1682, 1597, 1486, 1449, 1356, 1215, 1010, 831, 755, 689, 587. HRMS [$M+Na^+$]: calcd for $C_{18}H_{17}BrO_4Na$: 399.0208, found: 399.0208. HPLC analysis (IA column, λ = 254 nm, eluent: hexane/2-propanol 80/20, flow rate: 0.8 mL/min): t_R = 12.10 min (minor), 21.04 min (major).

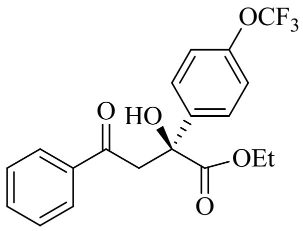

Compound **3d**: colorless oil, yield 92%, 76% ee, $[\alpha]_D^{25}$ -40.28 (c = 0.14, $CHCl_3$). ^1H NMR (400 MHz, $CDCl_3$): δ = 7.95-7.93 (m, 2H), 7.75-7.71 (m, 2H), 7.60-7.56 (m, 1H), 7.47-7.44 (m, 2H), 7.26-7.22 (m, 2H), 4.57 (s, 1H), 4.26 (q, J = 7.1 Hz, 2H), 4.04 (d, J = 17.7 Hz, 1H), 3.56 (d, J = 17.7 Hz, 1H), 1.25 (t, J = 7.1 Hz, 3H). ^{13}C NMR (101 MHz, $CDCl_3$): δ = 198.31, 173.65, 149.03, 149.01, 139.39, 136.24, 133.85, 128.74, 128.19, 126.87, 120.82, 76.09, 62.44, 49.04, 13.95. IR (KBr): ν = 3504, 3068, 2983, 2930, 1737, 1680, 1597, 1506, 1449, 1358, 1261, 1214, 1168, 1104, 1019, 852, 757, 689, 587. HRMS [$M+Na^+$]: calcd for $C_{19}H_{17}F_3O_5Na$: 405.0926, found: 405.0926. HPLC analysis (IA column, λ = 254 nm, eluent: hexane/2-propanol 80/20, flow rate: 0.8 mL/min): t_R = 9.86 min (minor), 15.48 min (major).

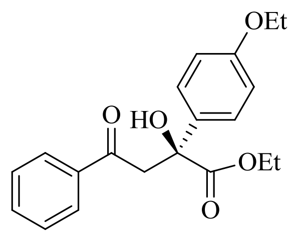

Compound **3e**: colorless solid, yield 85%, 49% ee, mp 77-79 °C, $[\alpha]_D^{25}$ -11.77 (c = 0.34, $CHCl_3$). ^1H NMR (400 MHz, $CDCl_3$): δ = 7.96-7.94 (m, 2H), 7.60-7.55 (m, 3H), 7.48-7.44 (m, 2H), 6.92-6.89 (m, 2H), 4.46 (s, 1H), 4.24 (q, J = 7.1 Hz, 2H), 4.07-4.01 (m, 3H), 3.56 (d, J = 17.7 Hz, 1H), 1.41 (t, J = 7.0 Hz, 3H), 1.24 (t, J = 7.1 Hz, 3H). ^{13}C NMR (101 MHz, $CDCl_3$): δ = 198.76, 174.21, 158.78, 136.46, 133.69, 132.61, 128.70, 128.19, 126.36, 114.38, 76.10, 63.49, 62.12, 48.93, 14.83, 14.03. IR (KBr): ν = 3521, 3466, 2984, 2936, 1732, 1683, 1606, 1509, 1450, 1346, 1245, 1204, 1177, 1106, 1042, 842, 761, 691, 575. HRMS [M+Na$^+$]: calcd for $C_{20}H_{22}O_5Na$: 365.1365, found: 365.1366. HPLC analysis (IA column, λ = 254 nm, eluent: hexane/2-propanol 80/20, flow rate: 0.8 mL/min): t_R = 13.04 min (minor), 21.42 min (major).

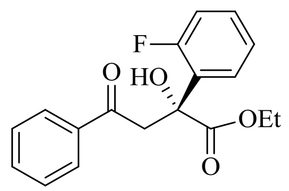

Compound **3f** : colorless oil, yield 81%, -41% ee (4S-pyBox ligand was used), $[\alpha]_D^{25}$ 16.00 (c = 0.20, $CHCl_3$). ^1H NMR (400 MHz, $CDCl_3$): δ = 7.94-7.54 (m, 5H), 7.45-7.21 (m, 4H), 4.56 (s, 1H), 4.27-4.20 (m, 2H), 4.03 (dd, J = 17.7, 12.9 Hz, 1H), 3.55 (dd, J = 17.7, 12.9 Hz, 1H), 1.23 (t, J = 7.1 Hz, 3H). ^{13}C NMR (101 MHz, $CDCl_3$): δ = 198.61, 198.22, 173.51, 143.07, 136.23, 133.87, 133.74, 131.22, 130.08, 128.76, 128.72, 128.53, 128.22, 128.09, 125.16, 123.92, 122.79, 76.43, 76.02, 62.47, 62.20, 49.00, 48.97, 14.02. IR (KBr): ν = 3507, 2981, 2929, 1731, 1682, 1597, 1449, 1357, 1263, 1215, 1179, 1061, 1002, 755, 689, 575. HRMS [M+Na$^+$]: calcd for $C_{18}H_{17}FO_4Na$: 339.1009, found: 339.1012. HPLC analysis (IA column, λ = 254 nm, eluent: hexane/2-propanol 80/20, flow rate: 0.8 mL/min): t_R = 9.09 min (major), 17.77 min (minor).

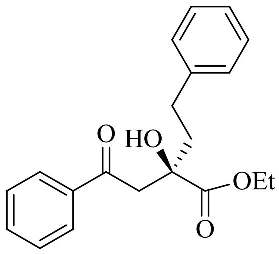

Compound **3g**: colorless oil, yield 93%, 77% ee, $[\alpha]_D^{25}$ -23.60 (c = 0.35, $CHCl_3$). ^1H NMR (400 MHz, $CDCl_3$): δ = 7.91-7.89 (m, 2H), 7.55-7.51 (m, 1H), 7.43-7.39 (m, 2H), 7.28-7.25 (m, 2H), 7.20-7.15 (m, 3H), 4.21 (q, J = 7.1 Hz, 2H), 4.05 (s, 1H), 3.57 (d, J = 17.4 Hz, 1H), 3.43 (d, J = 17.4 Hz, 1H), 2.93-2.84 (m, 1H), 2.61-2.53 (m, 1H), 2.11-2.07 (m, 2H), 1.24 (t, J = 7.1 Hz, 3H). ^{13}C NMR (101 MHz, $CDCl_3$): δ = 198.41, 175.31, 141.51, 136.59, 133.64, 128.71, 128.51, 128.48, 128.20, 126.06, 75.01, 61.81, 47.33, 41.37, 29.58, 14.26. IR (KBr): ν = 3517, 3061, 3027, 2927, 1730, 1682, 1598, 1451, 1362, 1216, 1122, 1089, 1018, 755, 691, 594, 498. HRMS $[M+Na^+]$: calcd for $C_{20}H_{22}O_4Na$: 349.1416, found: 349.1416. HPLC analysis (IA column, λ = 254 nm, eluent: hexane/2-propanol 80/20, flow rate: 0.8 mL/min): t_R = 10.70 min (minor), 13.27 min (major).

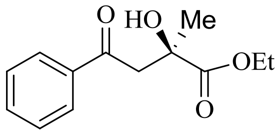

Compound **3h**: colorless oil, yield 88%, 60% ee, $[\alpha]_D^{25}$ -42.32 (c = 0.11, $CHCl_3$). ^1H NMR (400 MHz, $CDCl_3$): δ = 7.94-7.92 (m, 2H), 7.58-7.54 (m, 1H), 7.46-7.33 (m, 2H), 4.22 (q, J = 7.6 Hz, 2H), 4.04 (s, 1H), 3.65 (d, J = 17.6 Hz, 1H), 3.36 (d, J = 17.6 Hz, 1H), 1.51 (s, 3H), 1.23 (t, J = 7.6 Hz, 3H). ^{13}C NMR (101 MHz, $CDCl_3$): δ = 198.64, 175.85, 136.38, 133.56, 128.61, 128.08, 72.55, 61.55, 47.88, 26.41, 14.03. IR (KBr): ν = 3510, 2982, 2937, 1730, 1682, 1597, 1581, 1449, 1364, 1284, 1216, 1113, 1010, 757, 691, 627, 577. HRMS $[M+Na^+]$: calcd for $C_{13}H_{16}O_4Na$: 259.0946, found: 259.0956. HPLC analysis (OD-H column, λ = 254 nm, eluent: hexane/2-propanol 80/20, flow rate: 0.8 mL/min): t_R = 15.29 min (major), 16.99 min (minor).

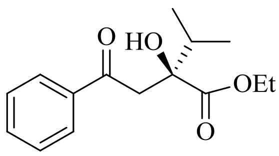

Compound **3i**: colorless oil, yield 91%, 56% ee, $[\alpha]_D^{25}$ -30.30 (*c* = 0.20, $CHCl_3$). ^1H NMR (400 MHz, $CDCl_3$): δ = 7.94-7.81 (m, 2H), 7.58-7.54 (m, 1H), 7.47-7.43 (m, 2H), 4.23 (q, *J* = 7.1 Hz, 2H), 3.80 (s, 1H), 3.48 (q, *J* = 17.3 Hz, 2H), 2.04-1.97 (m, 1H), 1.25 (t, *J* = 7.1 Hz, 3H), 1.06 (d, *J* = 6.9 Hz, 3H), 0.96 (d, *J* = 6.9 Hz, 3H). ^{13}C NMR (101 MHz, $CDCl_3$): δ = 198.82, 175.49, 136.81, 133.43, 128.60, 128.08, 61.52, 44.90, 35.76, 17.03, 16.55, 14.15. IR (KBr): ν = 3524, 3062, 2972, 2936, 1730, 1690, 1597, 1449, 1353, 1271, 1217, 1128, 1040, 1003, 756, 699, 587. HRMS [$M+Na^+$]: calcd for $C_{15}H_{20}O_4Na$: 287.1259, found: 287.1261. HPLC analysis (IA column, λ = 254 nm, eluent: hexane/2-propanol 80/20, flow rate: 0.8 mL/min): t_R = 8.36 min (minor), 9.74 min (major).

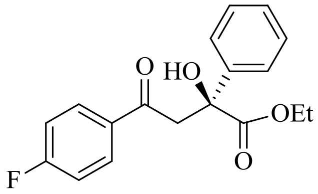

Compound **3j**: colorless solid, yield 88%, 75% ee, mp 61-62 °C, $[\alpha]_D^{25}$ -45.05 (*c* = 0.11, $CHCl_3$). ^1H NMR (400 MHz, $CDCl_3$): δ = 7.98-7.96 (m, 2H), 7.68-7.66 (m, 2H), 7.41-7.25 (m, 3H), 7.14-7.09 (m, 2H), 4.55 (s, 1H), 4.25 (q, *J* = 7.1 Hz, 2H), 4.02 (d, *J* = 17.6 Hz, 1H), 3.55 (d, *J* = 17.6 Hz, 1H), 1.24 (t, *J* = 7.1 Hz, 3H). ^{13}C NMR (101 MHz, $CDCl_3$): δ = 196.98, 174.00, 170.20, 167.39, 164.84, 140.63, 132.90, 132.88, 132.79, 130.98, 130.89, 128.54, 128.14, 125.10, 115.97, 115.78, 115.75, 115.56, 76.43, 62.30, 48.89, 13.99. IR (KBr): ν = 3516, 3110, 3000, 1720, 1676, 1599, 1590, 1450, 1389, 1352, 1273, 1212, 1160, 848, 784, 570. HRMS [$M+Na^+$]: calcd for $C_{18}H_{17}FO_4Na$: 339.1009, found: 339.1012. HPLC analysis (IA column, λ = 254 nm, eluent: hexane/2-propanol 80/20, flow rate: 0.8 mL/min): t_R = 11.21 min (minor), 20.41 min (major).

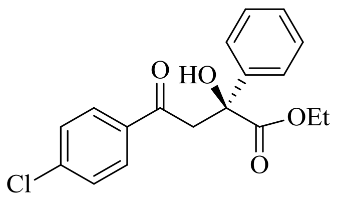

Compound **3k**: colorless solid, yield 91%, 60% ee, mp 56-58 °C, $[\alpha]_D^{25}$ -19.87 (*c* = 0.30, $CHCl_3$). ^{1}H NMR (400 MHz, $CDCl_3$): δ = 7.90-7.88 (m, 2H), 7.68-7.66 (m, 2H), 7.45-7.26 (m, 5H), 4.44 (s, 1H), 4.26 (q, *J* = 7.1 Hz, 2H), 4.00 (d, *J* = 17.6 Hz, 1H), 3.54 (d, *J* = 17.6 Hz, 1H), 1.26 (t, *J* = 7.1 Hz, 3H). ^{13}C NMR (101 MHz, $CDCl_3$): δ = 197.30, 173.91, 140.59, 140.26, 134.76, 129.61, 129.05, 128.55, 128.15, 125.08, 76.38, 62.33, 48.94, 14.01. IR (KBr): ν = 3410, 2986, 2922, 1731, 1681, 1588, 1401, 1379, 1205, 1055, 818, 726, 694. HRMS [M+Na$^+$]: calcd for $C_{18}H_{17}ClO_4Na$: 355.0713, found: 355.0716. HPLC analysis (IA column, λ = 254 nm, eluent: hexane/2-propanol 80/20, flow rate: 0.8 mL/min): t_R = 12.60 min (minor), 23.56 min (major).

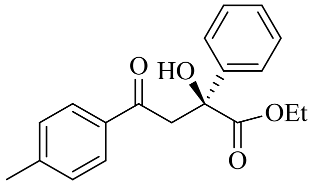

Compound **3l**: colorless solid, yield 92%, 60% ee, mp 74-76 °C, $[\alpha]_D^{25}$ -47.58 (*c* = 0.22, $CHCl_3$). ^{1}H NMR (400 MHz, $CDCl_3$): δ = 7.85-7.83 (m, 2H), 7.69-7.67 (m, 2H), 7.38-7.35 (m, 2H), 7.31-7.30 (m, 1H), 7.24-7.22 (m, 2H), 4.58 (s, 1H), 4.23 (q, *J* = 7.1 Hz, 2H), 4.04 (d, *J* = 17.7 Hz, 1H), 3.53 (d, *J* = 17.7 Hz, 1H), 2.39 (s, 3H), 1.22 (t, *J* = 7.1 Hz, 3H). ^{13}C NMR (101 MHz, $CDCl_3$): δ = 198.37, 174.06, 144.71, 140.81, 133.97, 129.40, 128.51, 128.35, 128.05, 125.15, 76.50, 62.15, 48.86, 21.71, 14.02. IR (KBr): ν = 3509, 3059, 2977, 2932, 1716, 1670, 1607, 1451, 1353, 1335, 1272, 1213, 1185, 1059, 944, 813, 710, 573. HRMS [M+Na$^+$]: calcd for $C_{19}H_{20}O_4Na$: 335.1259, found: 335.1260. HPLC analysis (IA column, λ = 254 nm, eluent: hexane/2-propanol 80/20, flow rate: 0.8 mL/min): t_R = 13.80 min (minor), 20.67 min (major).

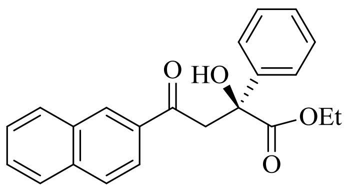

Compound **3m**: colorless solid, yield 93%, 59% ee, mp 78-79 ^{o}C, $[\alpha]_D^{25}$ -71.96 (*c* = 0.21, $CHCl_3$). ^{1}H NMR (400 MHz, $CDCl_3$): δ = 8.44 (s, 1H), 8.01-7.98 (m, 1H), 7.91-7.83 (m, 3H), 7.74-7.72 (m, 2H), 7.60-7.50 (m, 2H), 7.43-7.32 (m, 3H), 4.59 (s, 1H), 4.29-4.16 (m, 3H), 3.71 (d, *J* = 17.6 Hz, 1H), 1.25 (t, *J* = 7.1 Hz, 3H). ^{13}C NMR (101 MHz, $CDCl_3$): δ = 198.57, 174.09, 140.85, 135.88, 133.75, 132.44, 130.27, 129.67, 128.85, 128.63, 128.58, 128.14, 127.84, 126.97, 125.21, 123.58, 76.58, 62.27, 49.12, 14.07. IR (KBr): ν = 3524, 3055, 2923, 1725, 1658, 1626, 1597, 1468, 1449, 1366, 1209, 1209, 1187, 1122, 1056, 859, 817, 698, 583. HRMS [M+Na$^+$]: calcd for $C_{22}H_{20}O_4Na$: 371.1259, found: 371.1259. HPLC analysis (IA column, λ = 254 nm, eluent: hexane/2-propanol 80/20, flow rate: 0.8 mL/min): t_R = 14.86 min (minor), 24.72 min (major).

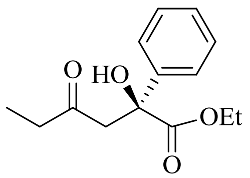

Compound **3n**: colorless oil, yield 81%, 49% ee, $[\alpha]_D^{25}$ -14.82 (*c* = 0.11, $CHCl_3$). ^{1}H NMR (400 MHz, $CDCl_3$): δ = 7.59-7.57 (m, 2H), 7.37-7.26 (m, 3H), 4.44 (s, 1H), 4.22 (q, *J* = 7.1 Hz, 2H), 3.52 (d, *J* = 17.4 Hz, 1H), 2.98 (d, *J* = 17.4 Hz, 1H), 3.00-2.96 (m, 2H), 1.24 (t, *J* = 7.1 Hz, 3H), 1.07 (t, *J* = 7.3 Hz, 3H). ^{13}C NMR (101 MHz, $CDCl_3$): δ = 210.43, 173.92, 140.61, 128.44, 127.99, 124.94, 76.39, 62.22, 51.87, 36.71, 13.97, 7.41. IR (KBr): ν = 3483, 2980, 2938, 1736, 1719, 1599, 1448, 1367, 1250, 1203, 1131, 1093, 1027, 728, 700, 581. HRMS [M+Na$^+$]: calcd for $C_{14}H_{18}O_4Na$: 273.1103, found: 273.1156. HPLC analysis (OD-H column, λ = 254 nm, eluent: hexane/2-propanol 80/20, flow rate: 0.8 mL/min): t_R = 7.62 min (major), 9.87 min (minor).

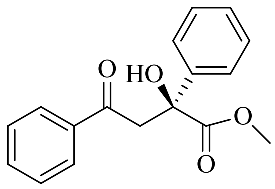

Compound **3o**: colorless solid, yield 93%, 47% ee, mp 67-69 °C, $[\alpha]_D^{25}$ -31.23 (*c* = 0.20, $CHCl_3$). ^1H NMR (400 MHz, $CDCl_3$): δ = 7.94-7.91 (m, 2H), 7.68-7.66 (m, 2H), 7.57-7.53 (m, 1H), 7.44-7.29 (m, 5H), 4.57 (s, 1H), 4.06 (d, *J* = 17.7 Hz, 1H), 3.74 (s, 3H), 3.57 (d, *J* = 17.7 Hz, 1H). ^{13}C NMR (101 MHz, $CDCl_3$): δ = 198.79, 174.58, 140.62, 136.31, 133.83, 128.75, 128.60, 128.25, 128.18, 125.14, 76.54, 53.12, 49.00. IR (KBr): ν = 3515, 3062, 2950, 1729, 1665, 1594, 1448, 1268, 1213, 1062, 1002, 948, 753, 689, 581. HRMS [M+Na$^+$]: calcd for $C_{17}H_{16}O_4Na$: 307.0942, found: 307.0943. HPLC analysis (IA column, λ = 254 nm, eluent: hexane/2-propanol 80/20, flow rate: 0.8 mL/min): t_R = 11.94 min (minor), 16.49 min (major).

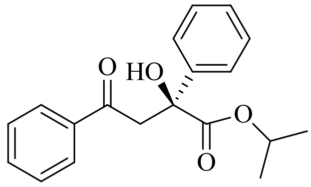

Compound **3p**: colorless solid, yield 91%, 71% ee, mp 71-72 °C, $[\alpha]_D^{25}$ -16.01 (*c* = 0.20, $CHCl_3$). ^1H NMR (400 MHz, $CDCl_3$): δ = 7.92-7.90 (m, 2H), 7.70-7.67 (m, 2H), 7.53-7.50 (m, 1H), 7.42-7.27 (m, 5H), 5.13-5.03 (m, 1H), 4.51 (s, 1H), 4.02 (d, *J* = 17.7 Hz, 1H), 3.55 (d, *J* = 17.7 Hz, 1H), 1.21 (d, *J* = 6.3 Hz, 3H), 1.17 (d, *J* = 6.3 Hz, 3H). ^{13}C NMR (101 MHz, $CDCl_3$): δ = 198.36, 173.53, 141.01, 136.53, 133.66, 128.72, 128.48, 128.17, 128.04, 125.20, 76.35, 69.95, 49.01, 21.57, 21.50. IR (KBr): ν = 3495, 2981, 2933, 1722, 1684, 1597, 1580, 1449, 1354, 1265, 1213, 1110, 1058, 1002, 759, 699, 571. HRMS [M+Na$^+$]: calcd for $C_{17}H_{16}O_4Na$: 335.1252, found: 335.1260. HPLC analysis (IA column, λ = 254 nm, eluent: hexane/2-propanol 80/20, flow rate: 0.8 mL/min): t_R = 9.9 min (minor), 16.88 min (major).

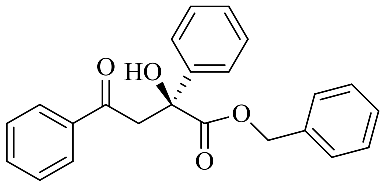

Compound **3q**: colorless solid, yield 89%, 67% ee, mp 90-91 ^{o}C, $[\alpha]_D^{25}$ -23.50 (c = 0.20, $CHCl_3$). ^{1}H NMR (400 MHz, $CDCl_3$): δ = 7.91-7.88 (m, 2H), 7.66-7.63 (m, 2H), 7.55-7.51 (m, 1H), 7.43-7.18 (m, 10H), 5.21-5.15 (m, 2H), 4.58 (s, 1H), 4.05 (d, J = 17.7 Hz, 1H), 3.57 (d, J = 17.7 Hz, 1H). ^{13}C NMR (101 MHz, $CDCl_3$): δ = 198.72, 173.97, 140.54, 136.39, 135.33, 133.81, 128.75, 128.58, 128.51, 128.31, 128.26, 128.21, 128.17, 125.28, 76.60, 67.83, 48.85. IR (KBr): ν = 3529, 3058, 3031, 2927, 1739, 1674, 1597, 1452, 1351, 1266, 1217, 1193, 1057, 995, 753, 688, 572. HRMS [M+Na$^+$]: calcd for $C_{23}H_{20}O_4Na$: 383.1024, found: 383.1256. HPLC analysis (IA column, λ = 254 nm, eluent: hexane/2-propanol 80/20, flow rate: 0.8 mL/min): t_R = 17.17 min (minor), 25.04 min (major).

Proof of absolute stereochemistry. The spectral data for Compound **3g** are consistent with those reported in the literature[4-5]. Moreover, as summarized in the table 1, the optical rotation measurements correspond to the (*R*) enantiomer being formed as the major enantiomer with chiral ligand **6a**.

Table 1. Comparison of Specific Rotation Values

Entry	study	Product ee(%)	Specific rotation $[(\alpha)_D]$	absolute configuration
1	Bolm (ref 4)	96	–28.5 (c = 0.80, $CHCl_3$)	*R*
2	Hoveyda (ref 5)	86	-26.5 (c = 1.00, $CHCl_3$)	*R*
2	This Study	77	–23.6 (c = 0.35, $CHCl_3$)	*R*

References

1. H. He, X.-J. Zheng, Y. Li, L.-X. Dai and S.-L. You, *Org. Lett.*, **2007**, *9*, 4339-4341.
2. H.-J. Zheng, W.-B. Chen, Z.-J. Wu, J.-G. Deng, W.-Q. Lin, W.-C. Yuan and X.-M. Zhang, *Chem. Eur. J.*, **2008**, *14*, 9864-9867.
3. M. Hayashi and S. Nakamura, *Angew. Chem. Int. Ed.*, **2011**, *50*, 2249-2252.
4. M. Langner and C. Bolm, *Angew. Chem. Int. Ed.*, **2004**, *43*, 5984-5987.
5. L. C. Akullian, M. L. Snapper and A. H. Hoveyda, *J. Am. Chem. Soc.*, **2006**, *128*, 6532-6533.

3. ^{1}H and ^{13}C NMR spectra for compound 3

^{1}H NMR of **3a**

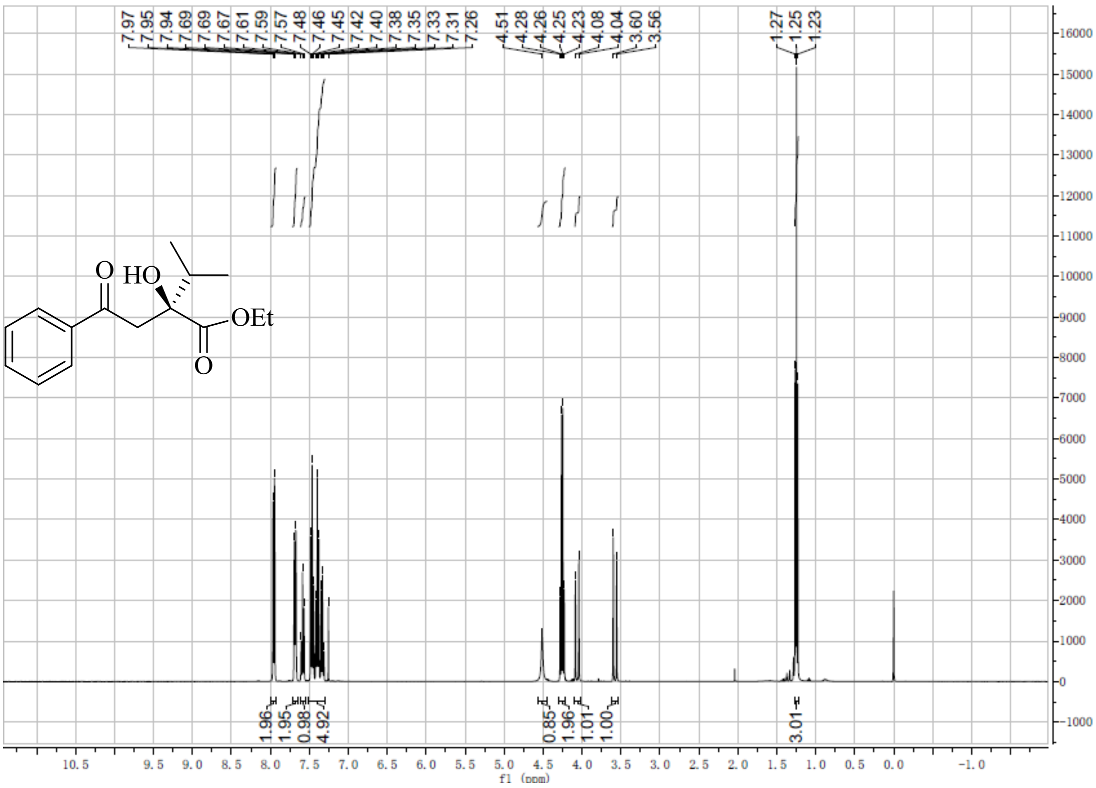

^{13}C NMR of **3a**

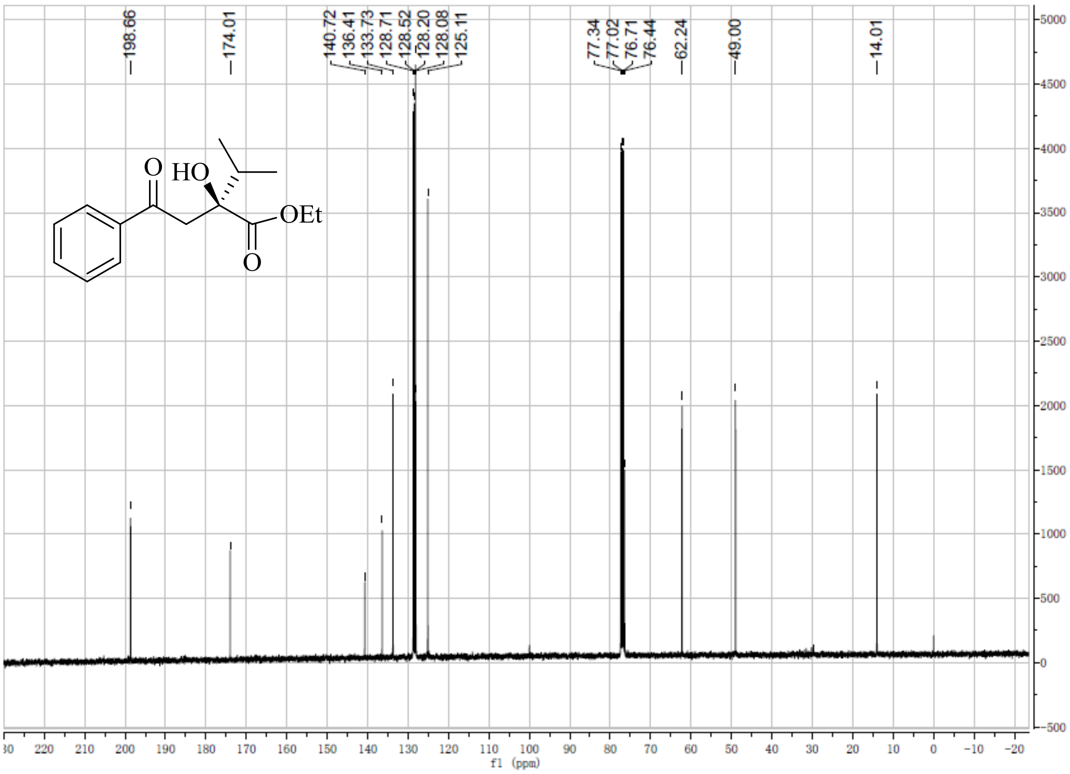

^{1}H NMR of **3b**

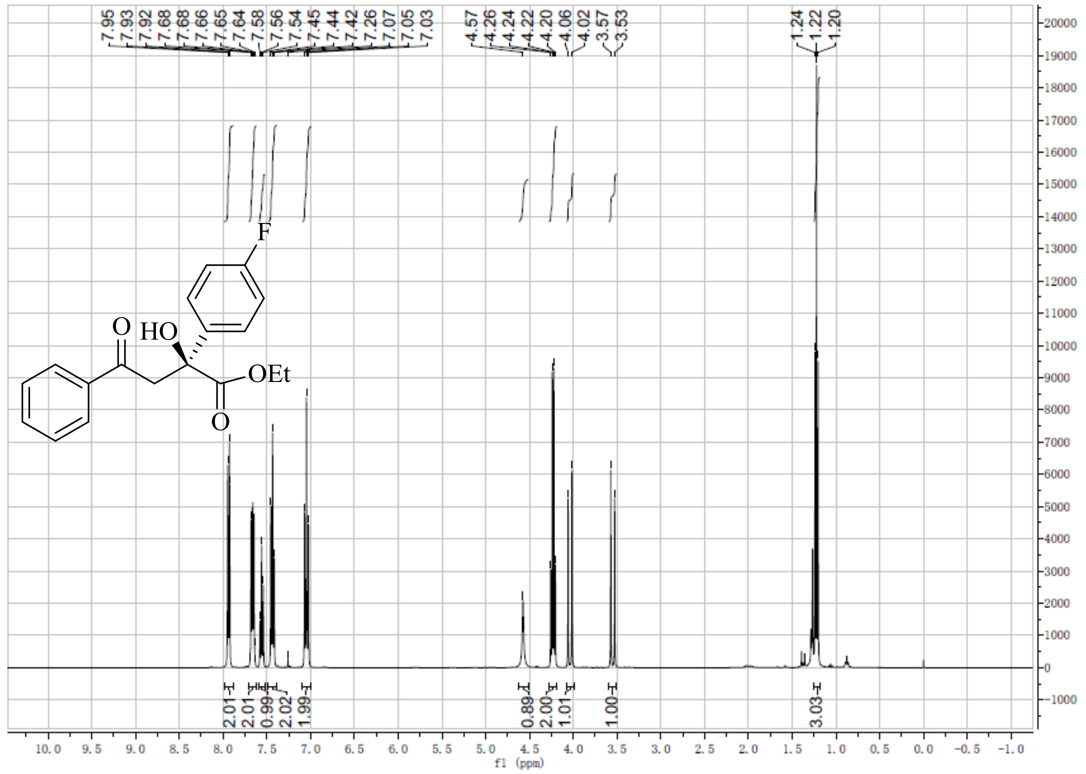

^{13}C NMR of **3b**

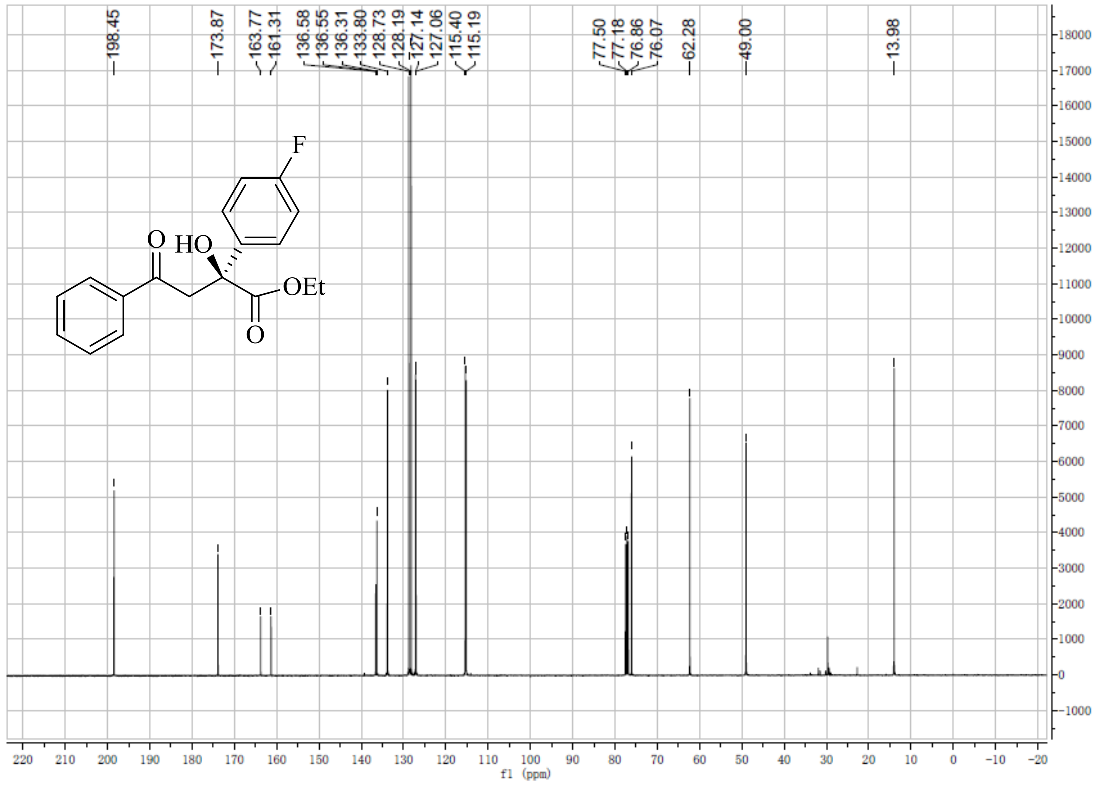

^{1}H NMR of **3c**

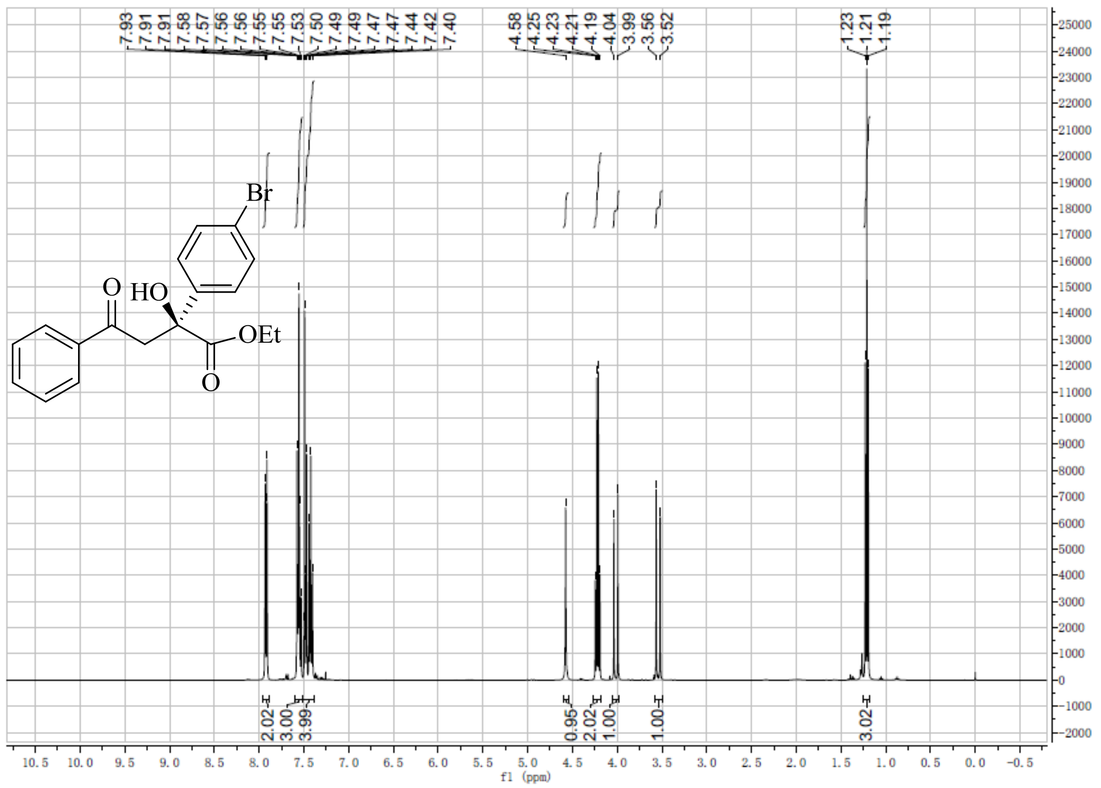

^{13}C NMR of **3c**

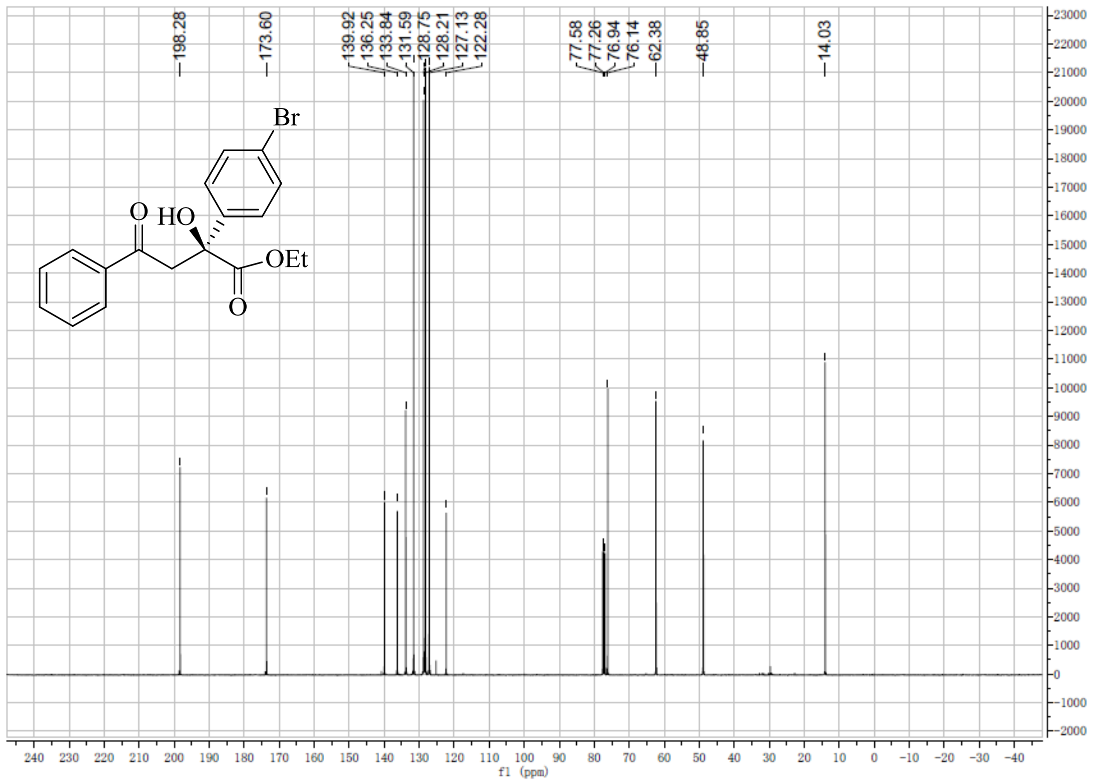

^{1}H NMR of **3d**

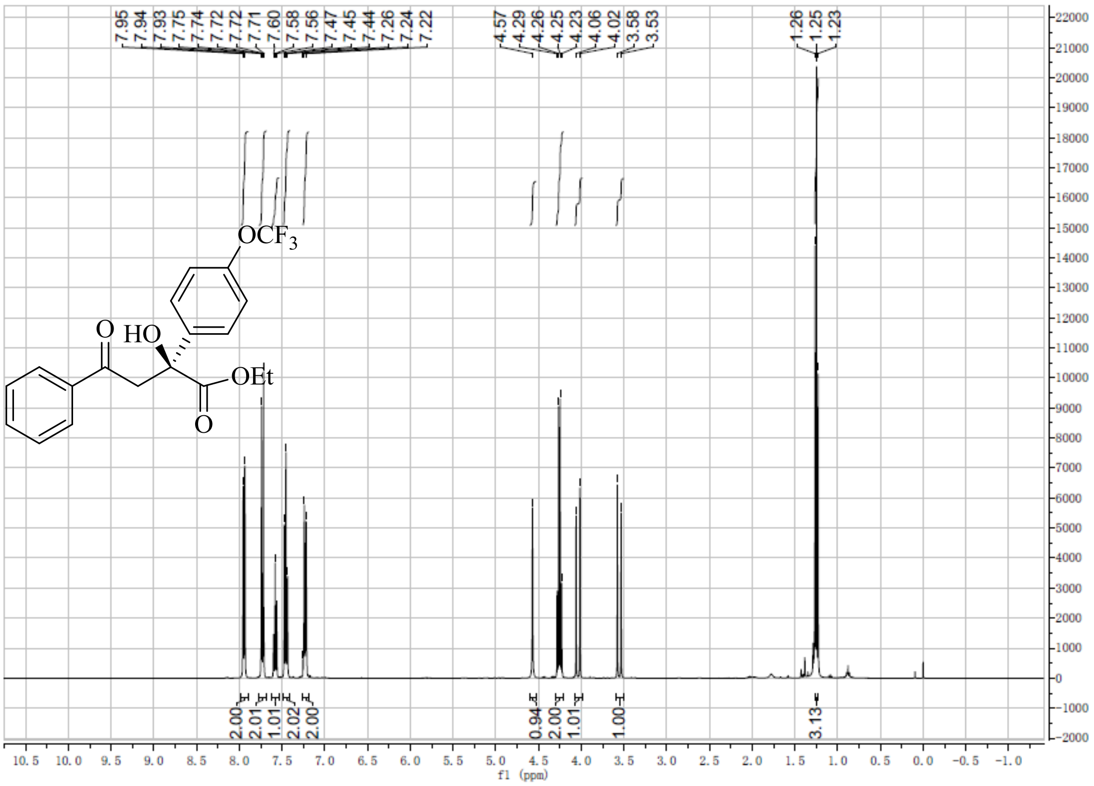

^{13}C NMR of **3d**

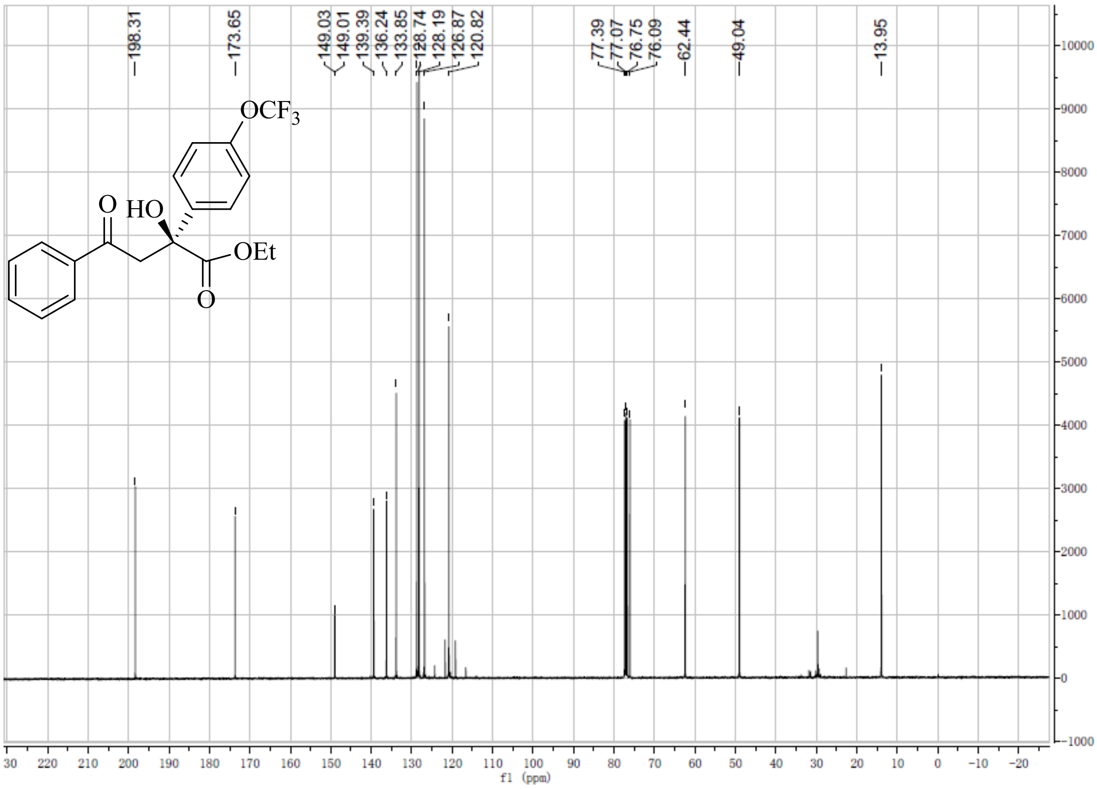

^{1}H NMR of **3e**

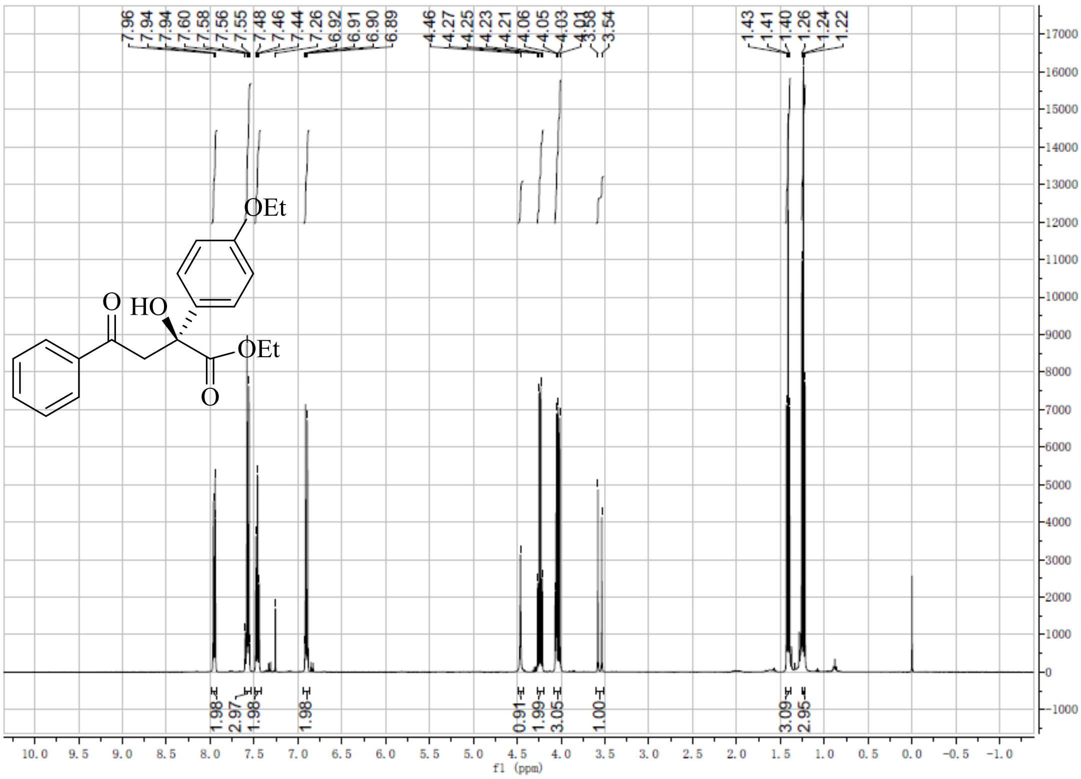

^{13}C NMR of **3e**

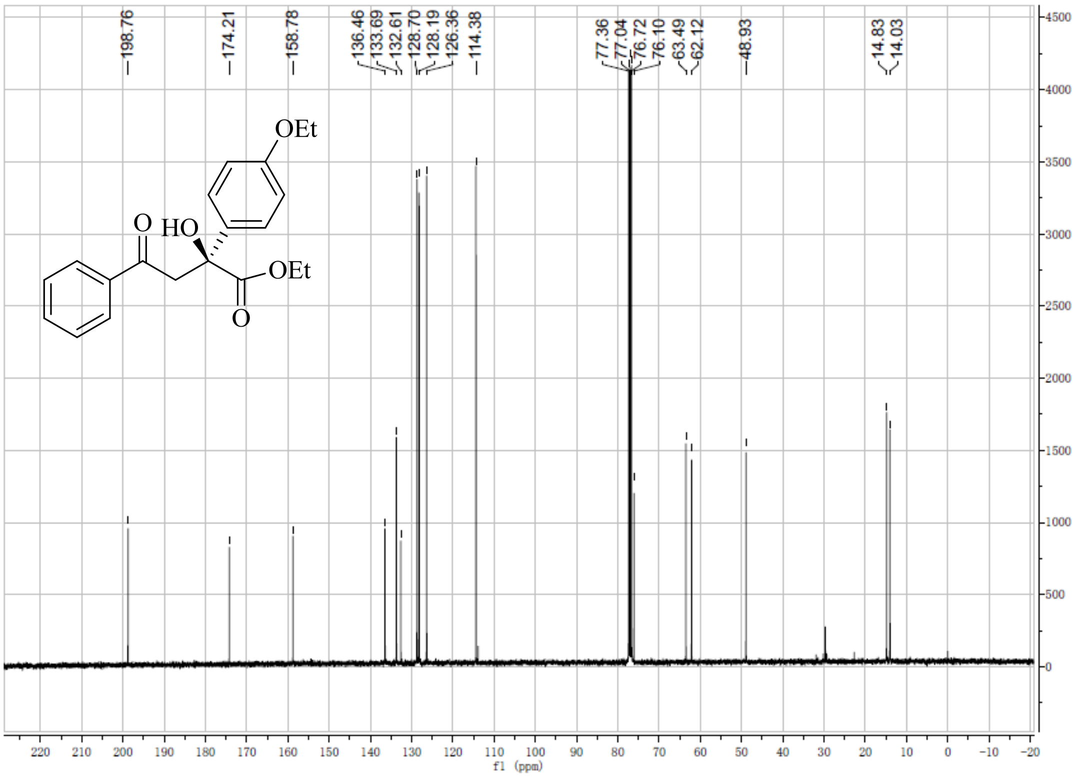

^{1}H NMR of **3f**

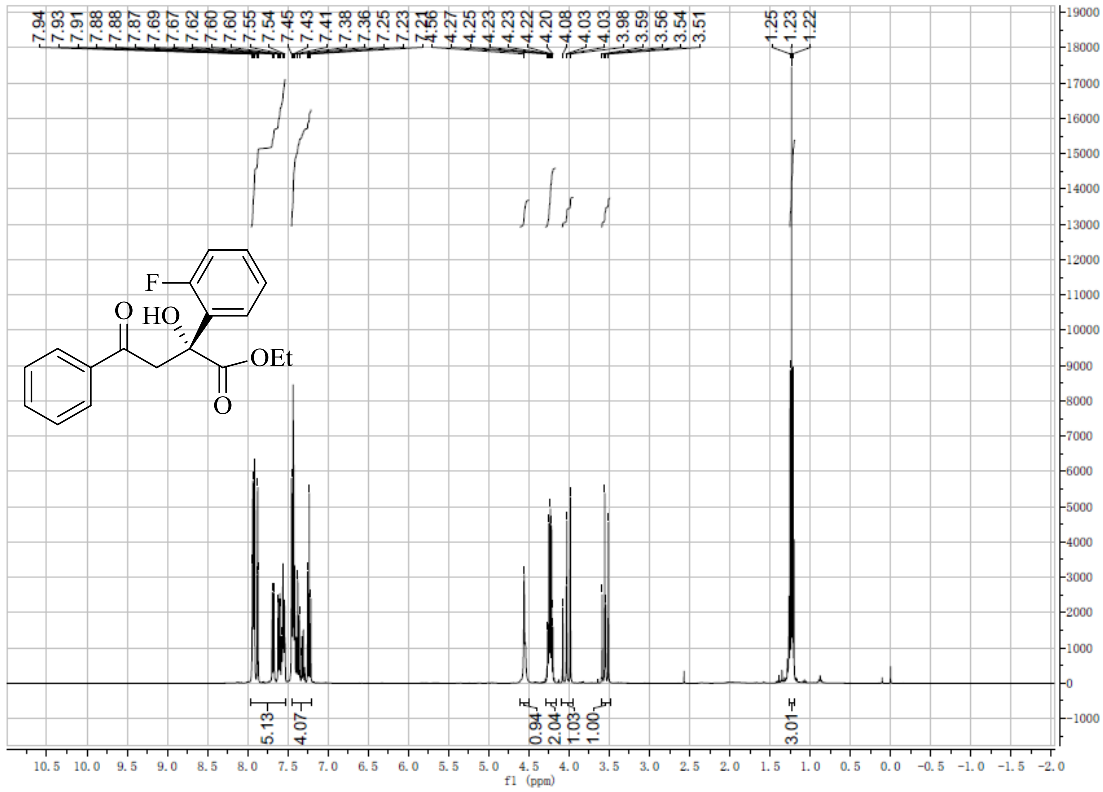

^{13}C NMR of **3f**

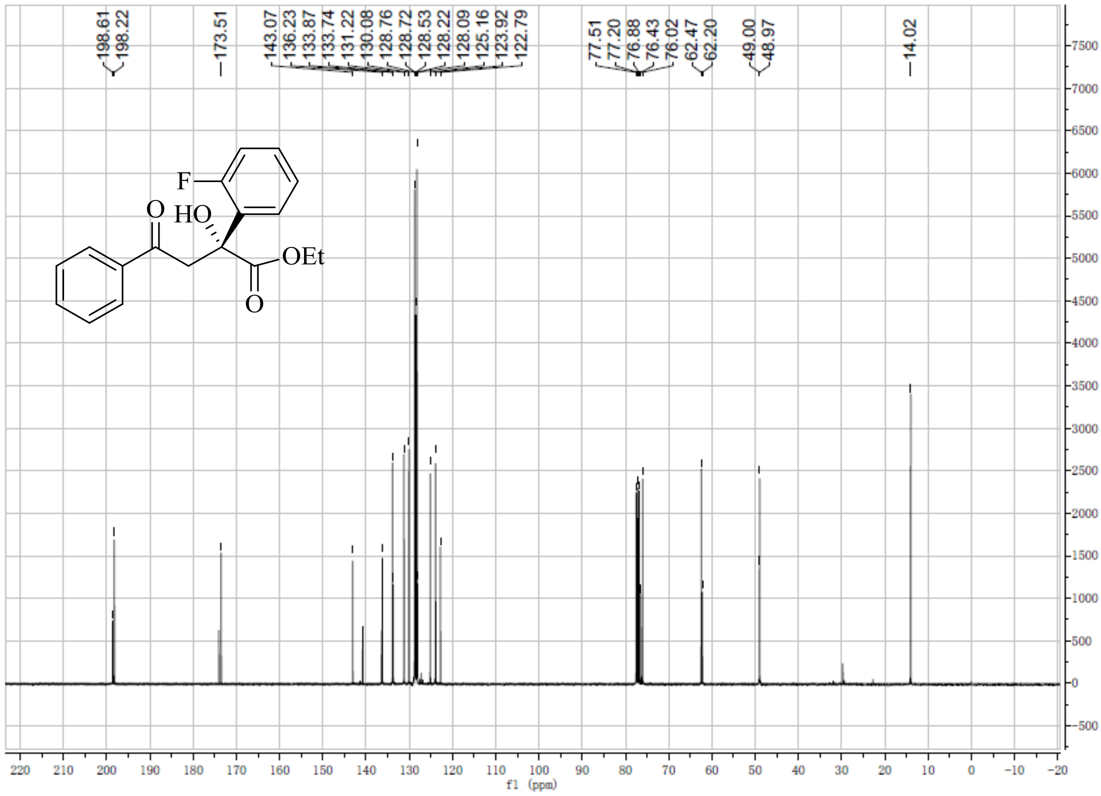

^{1}H NMR of **3g**

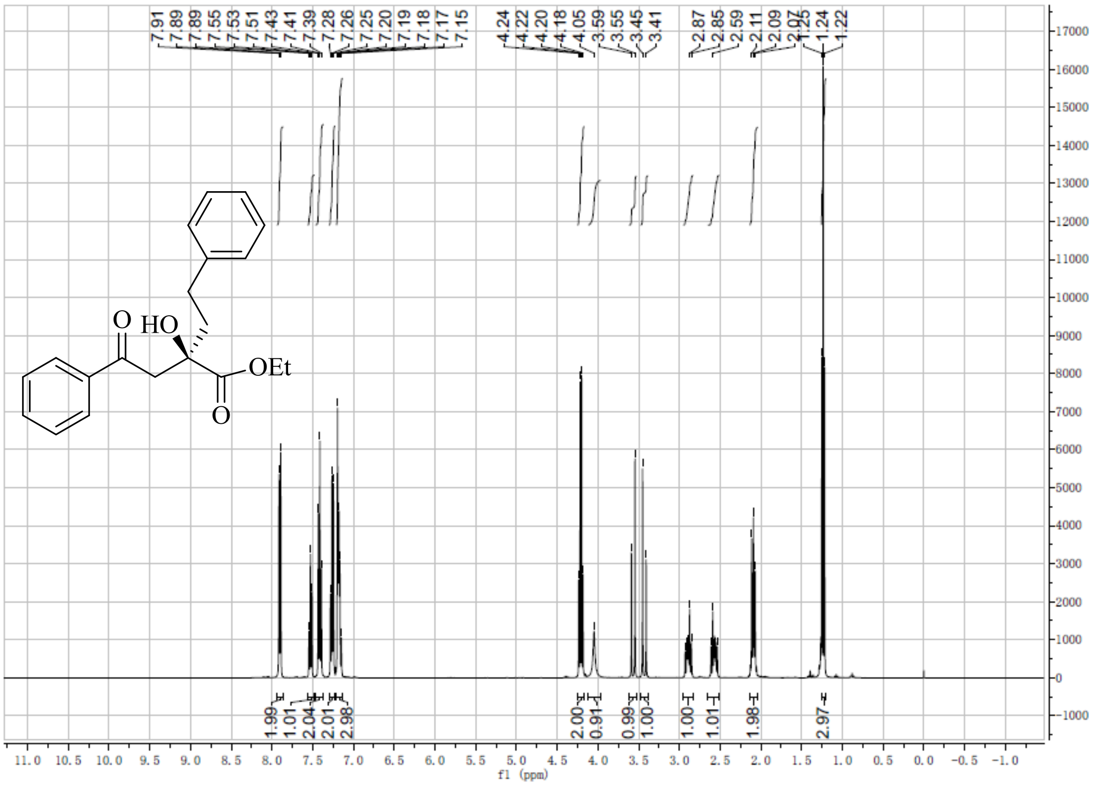

^{13}C NMR of **3g**

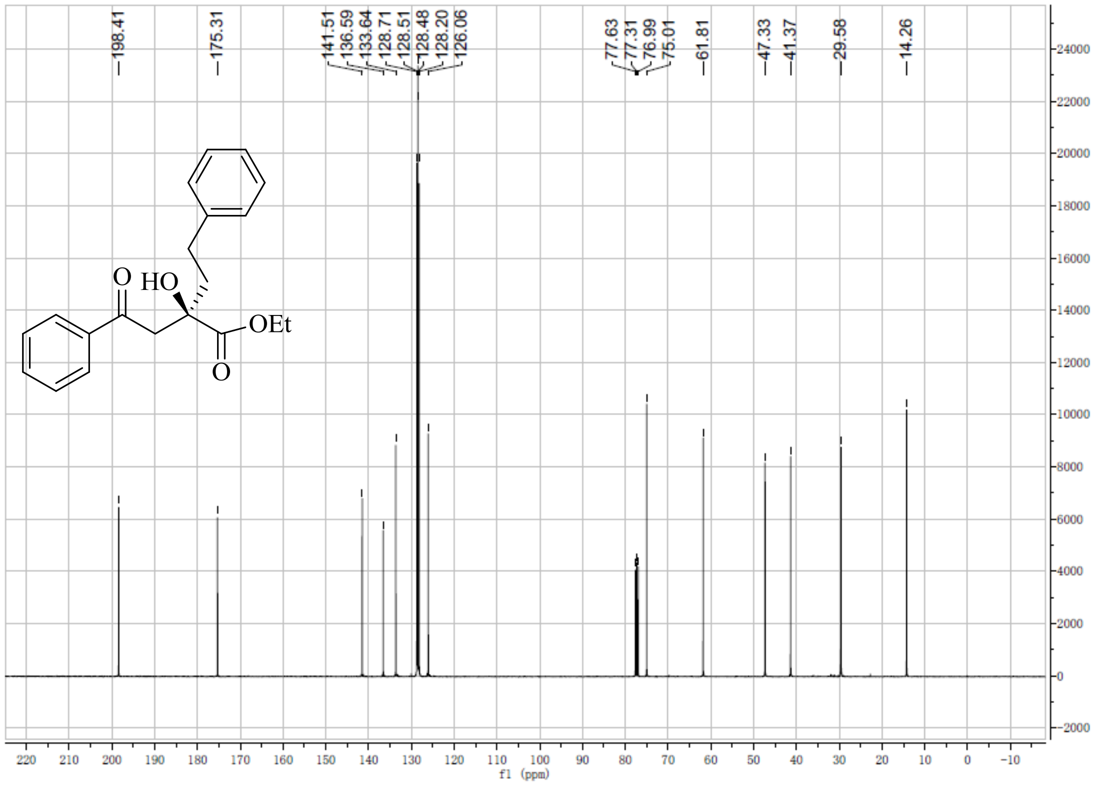

^{1}H NMR of **3h**

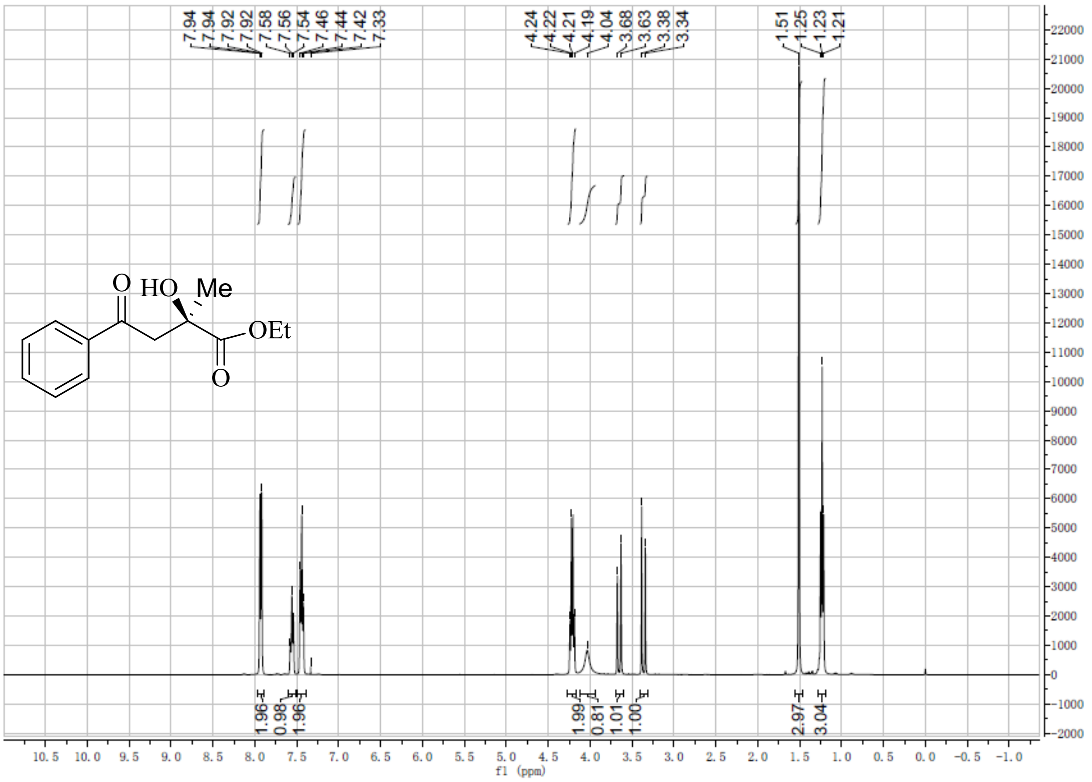

^{13}C NMR of **3h**

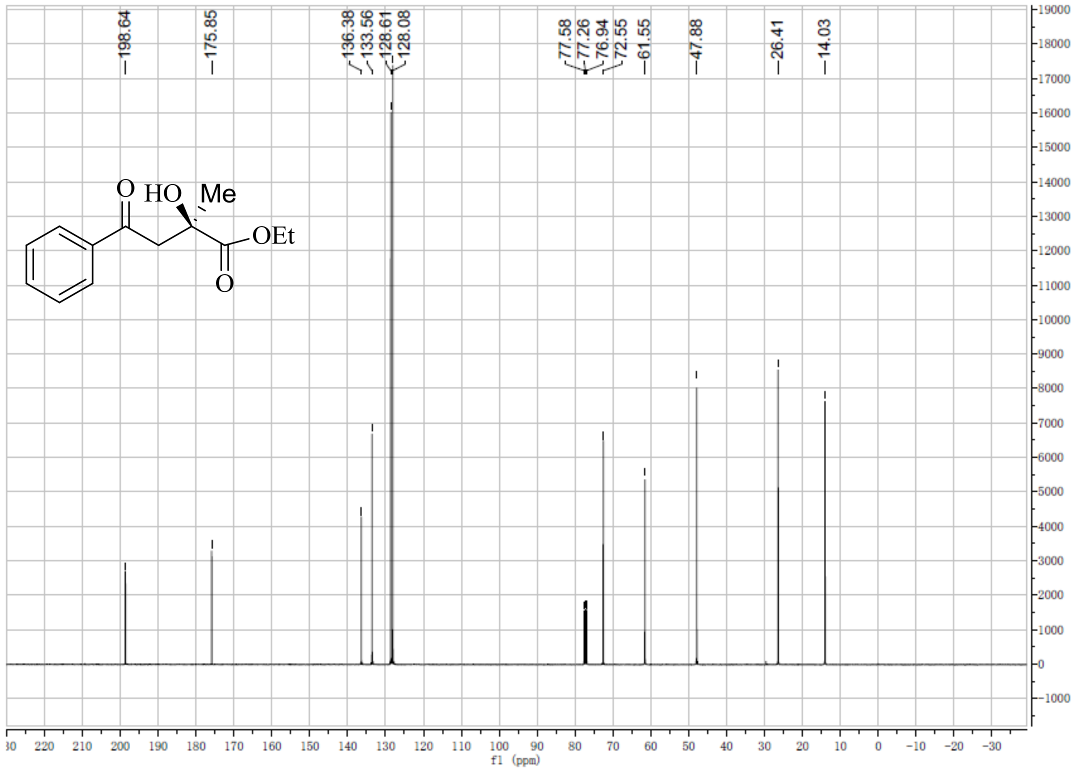

^{1}H NMR of **3i**

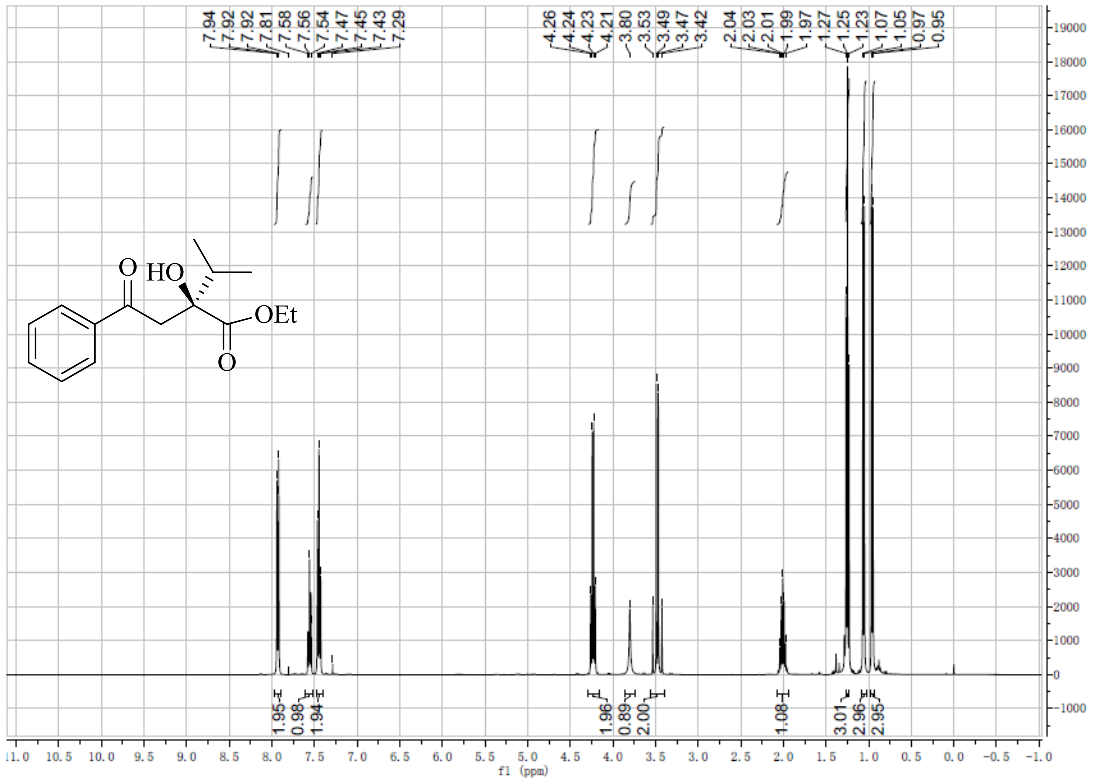

^{13}C NMR of **3i**

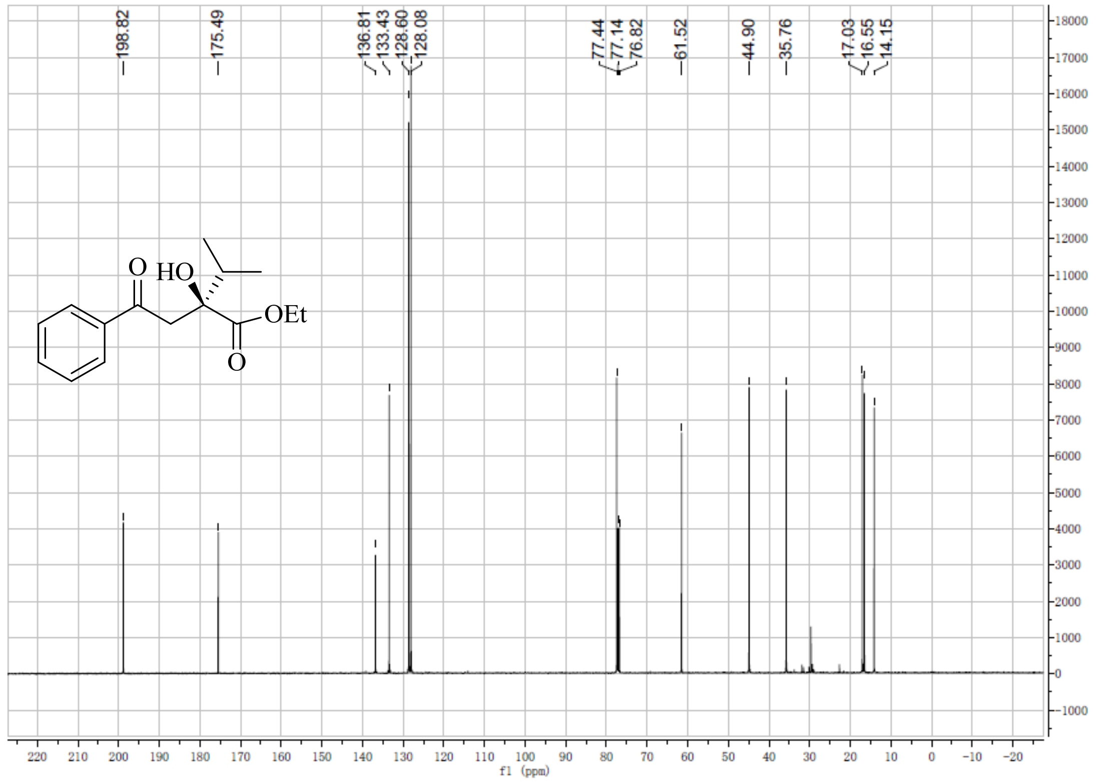

^{1}H NMR of **3j**

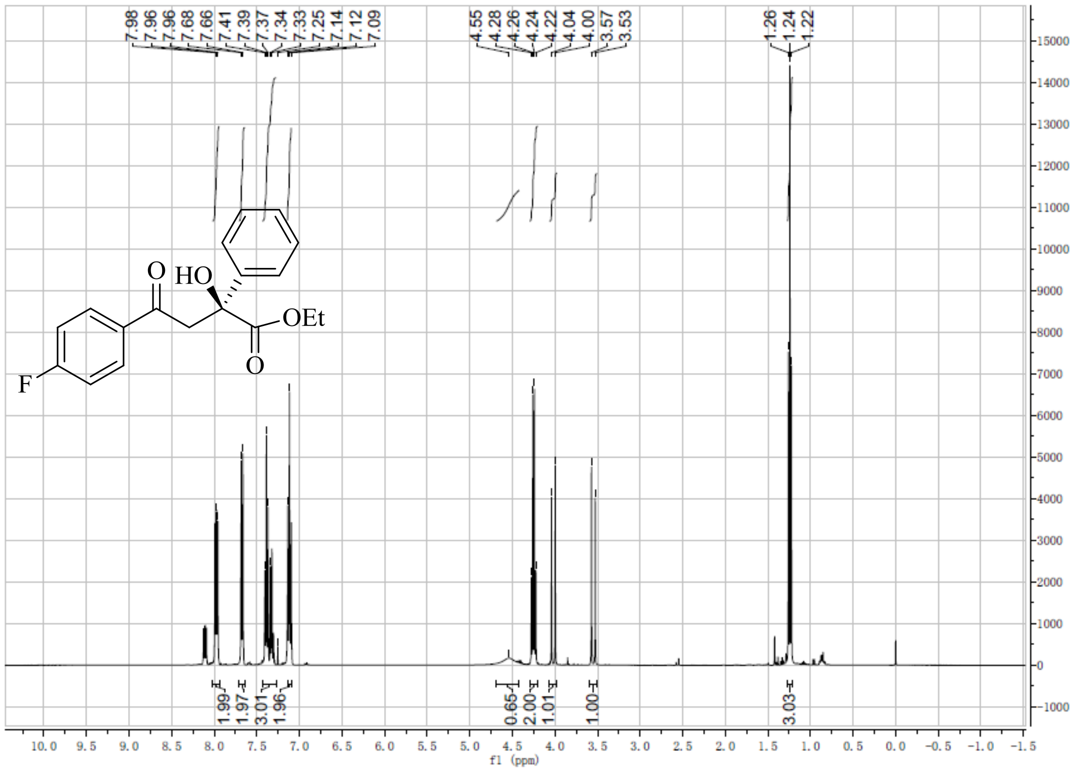

^{13}C NMR of **3j**

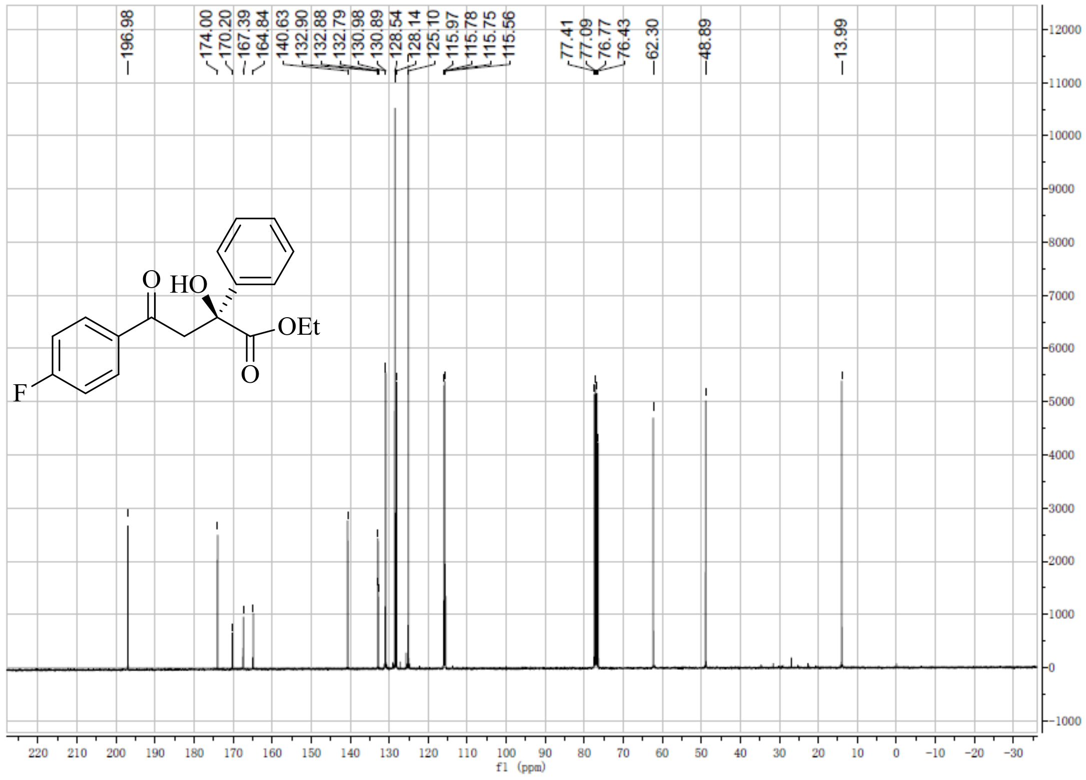

^{1}H NMR of **3k**

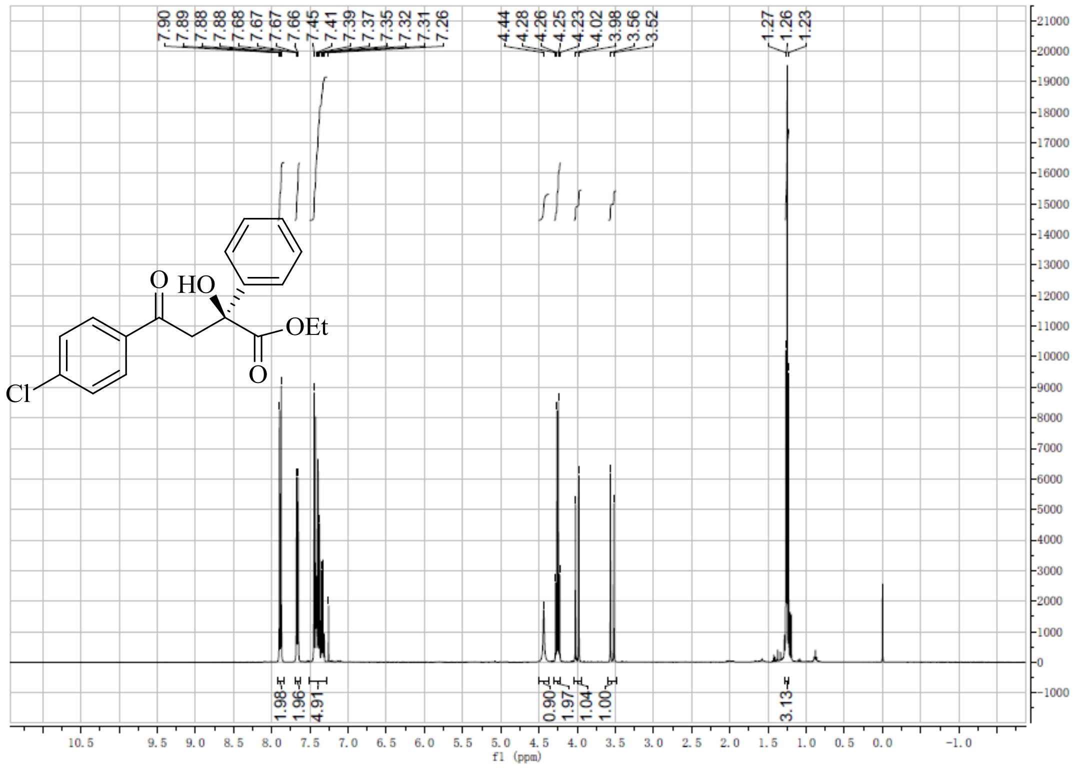

^{13}C NMR of **3k**

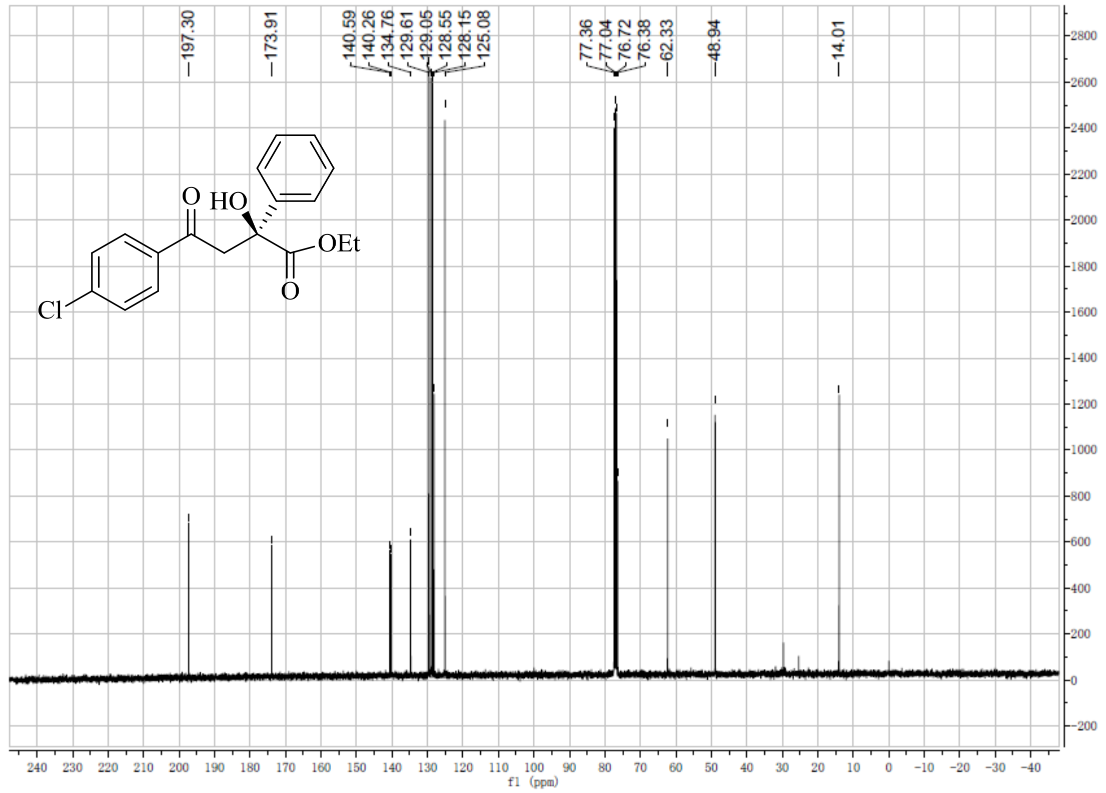

^{1}H NMR of **3l**

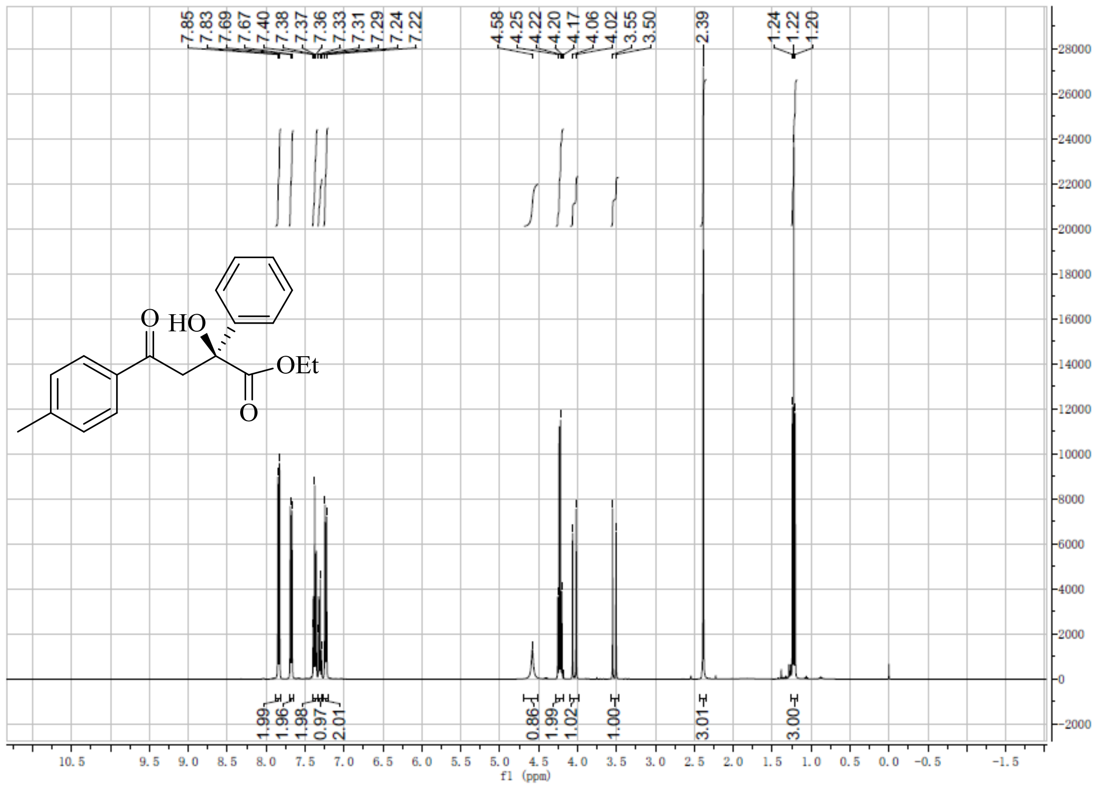

^{13}C NMR of **3l**

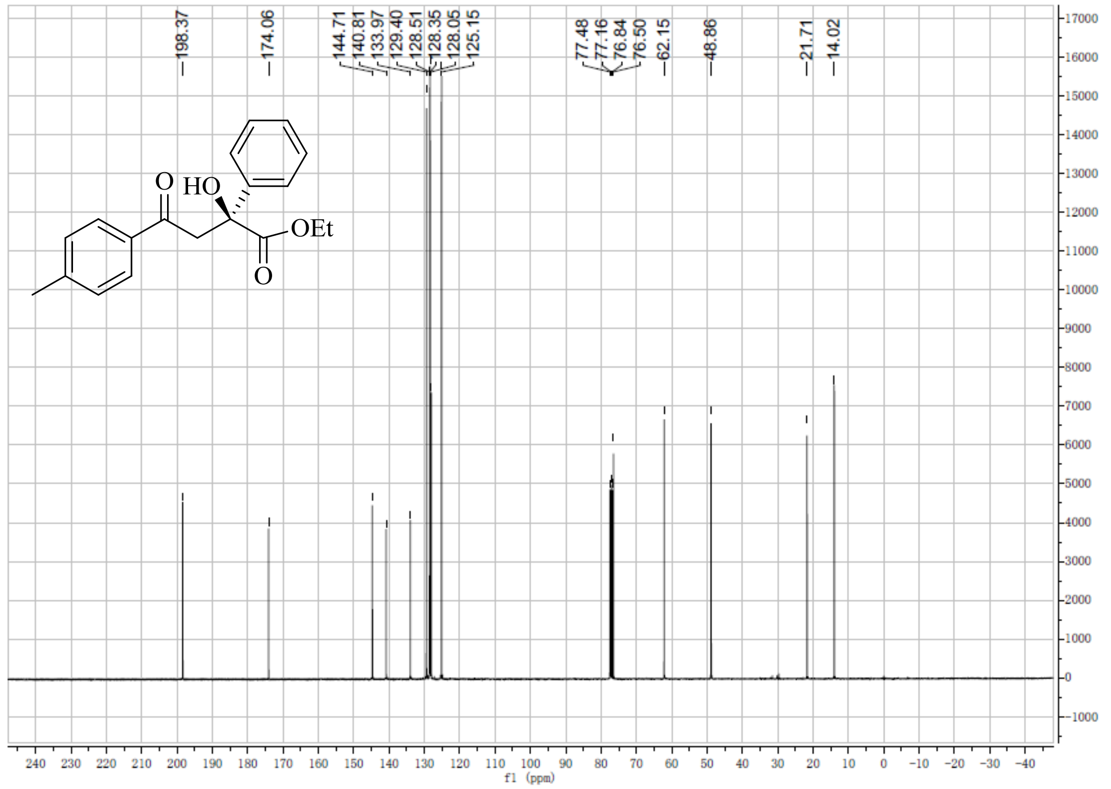

^{1}H NMR of **3m**

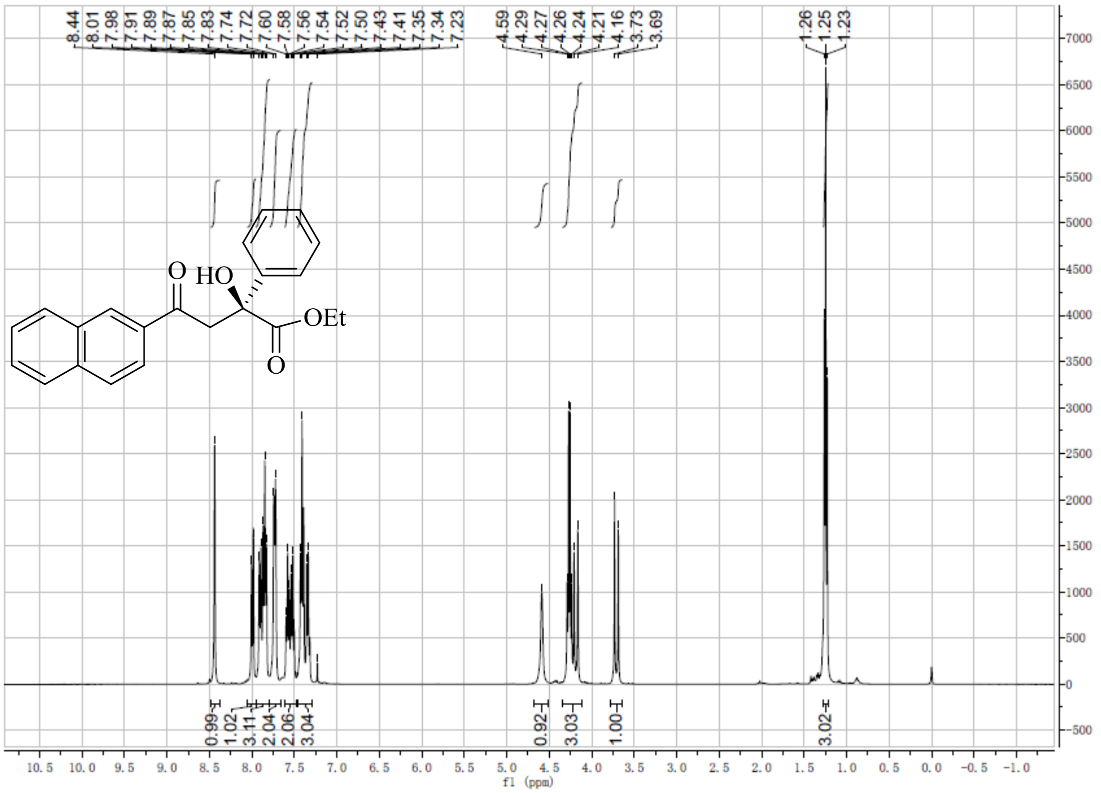

^{13}C NMR of **3m**

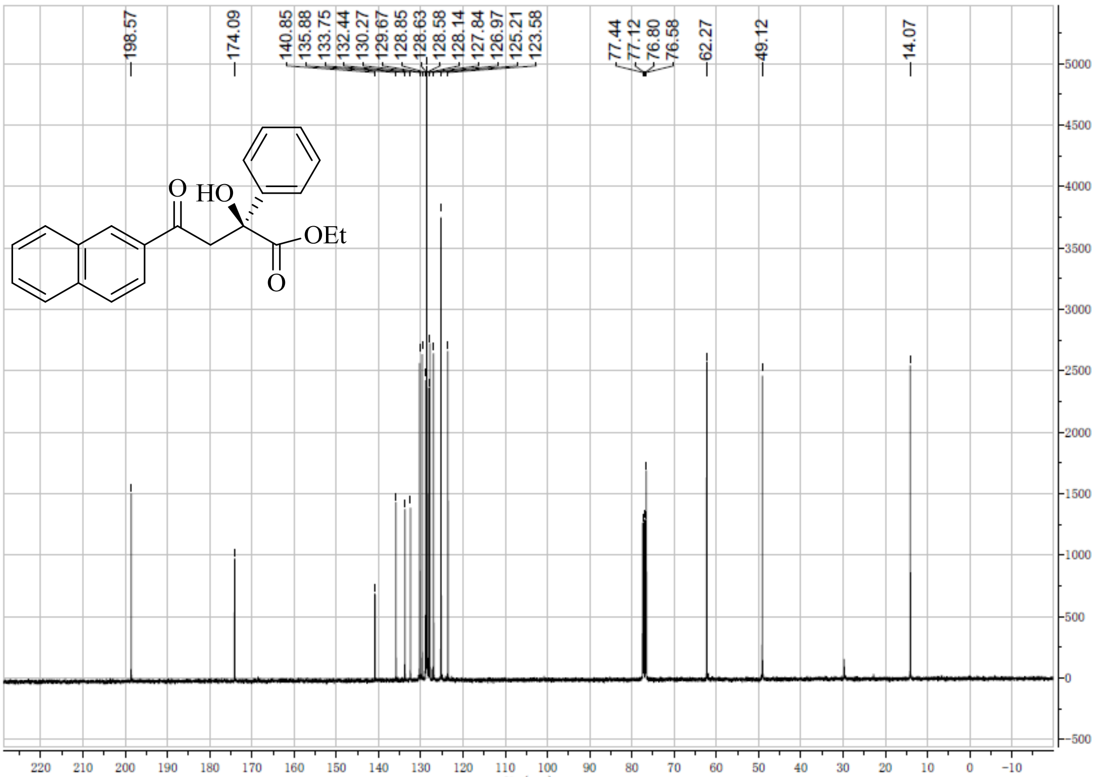

^{1}H NMR of **3n**

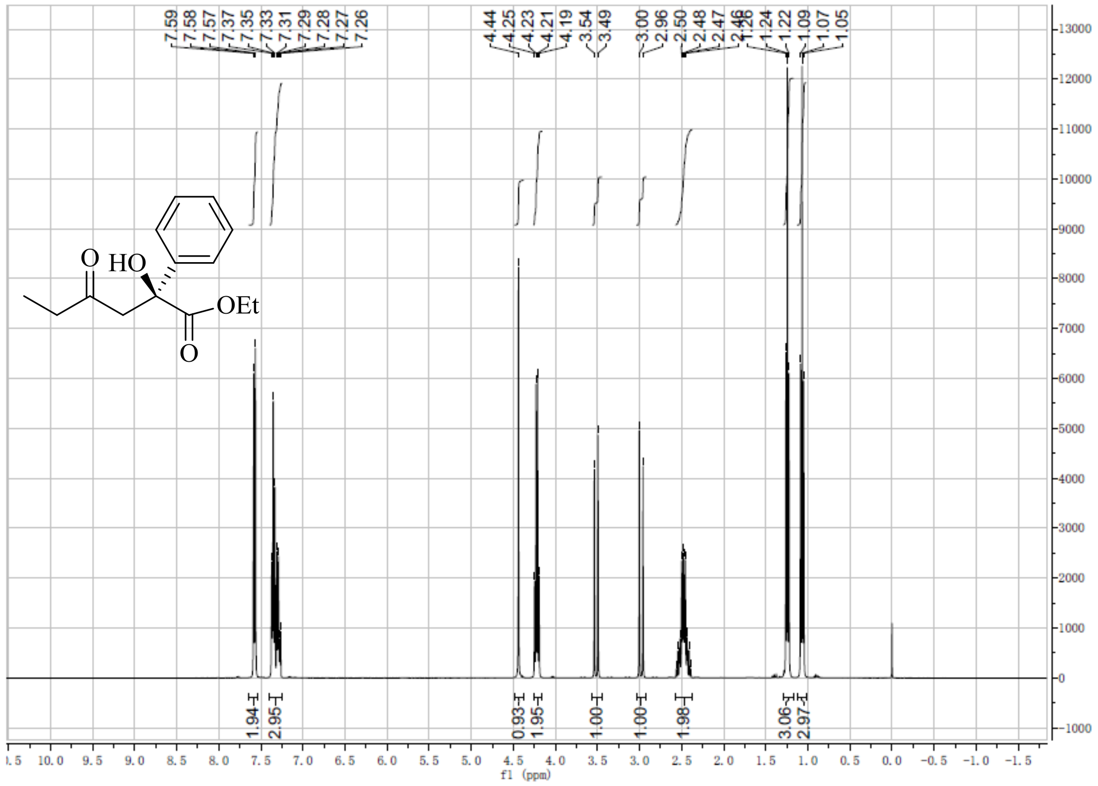

^{13}C NMR of **3n**

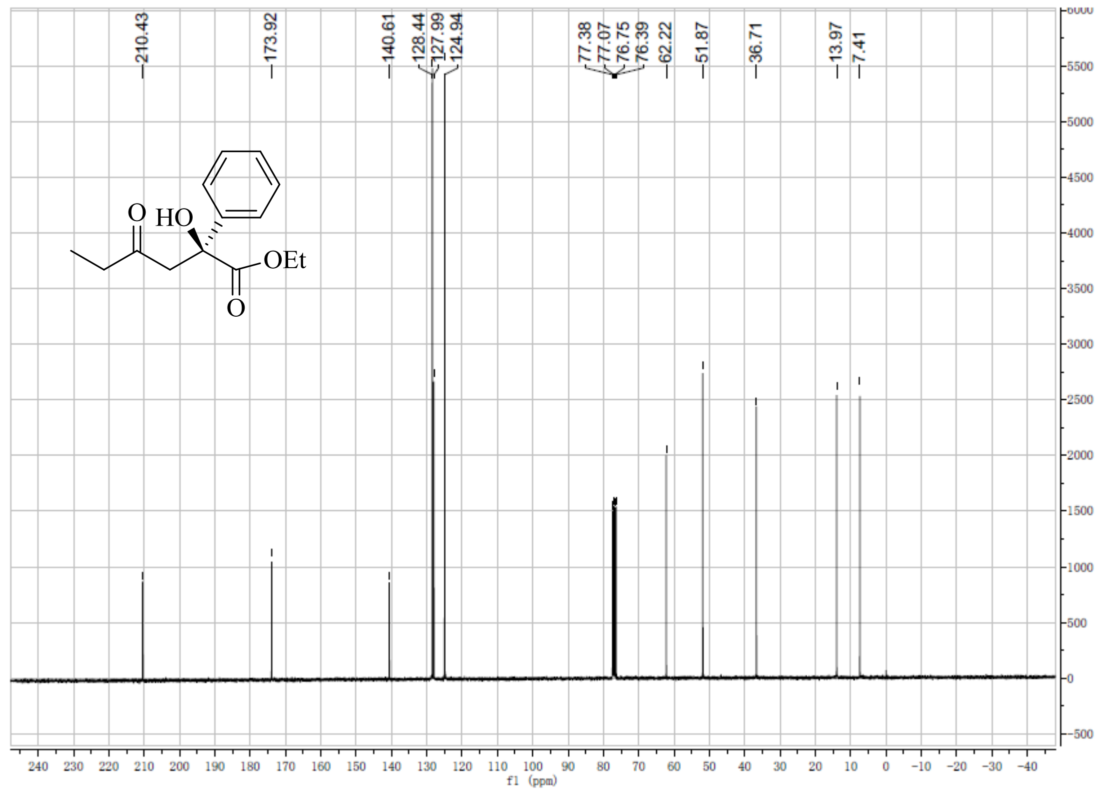

^{1}H NMR of **3o**

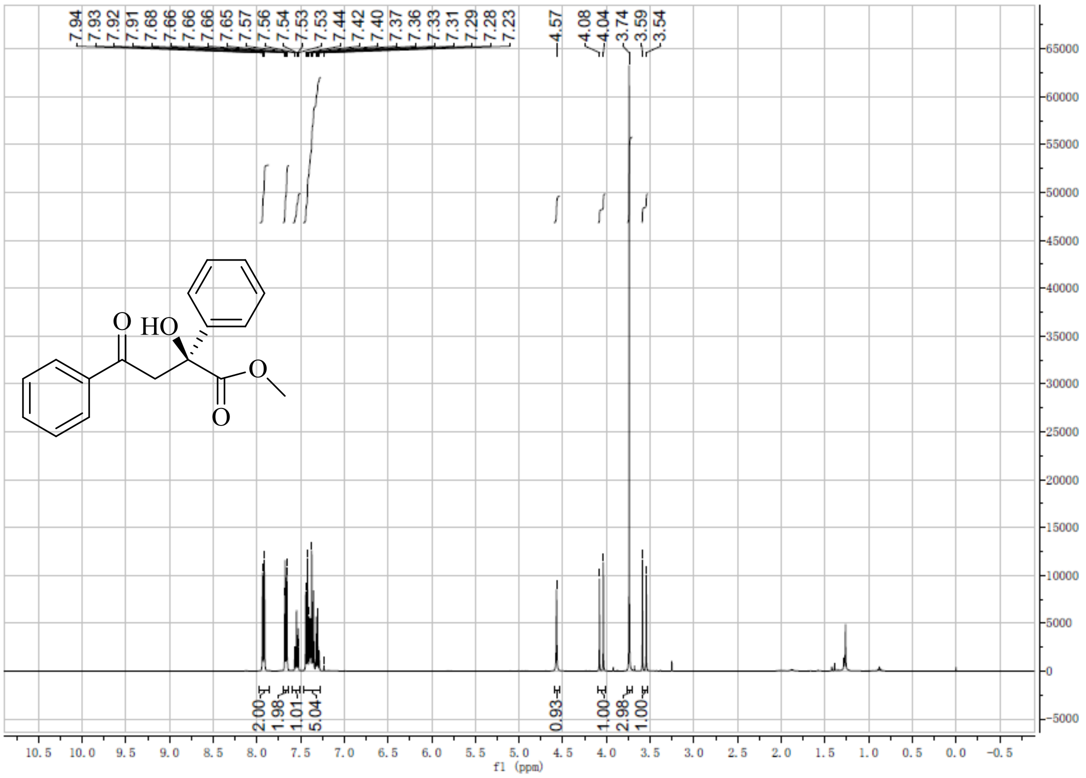

^{13}C NMR of **3o**

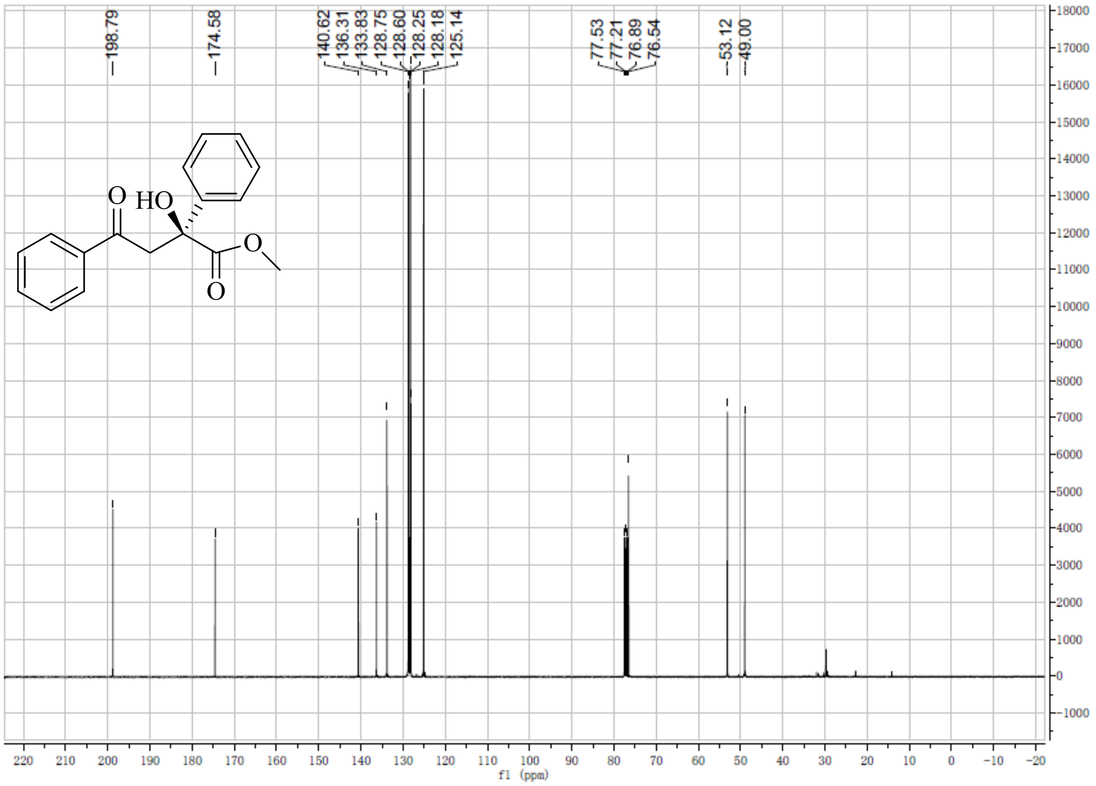

^{1}H NMR of **3p**

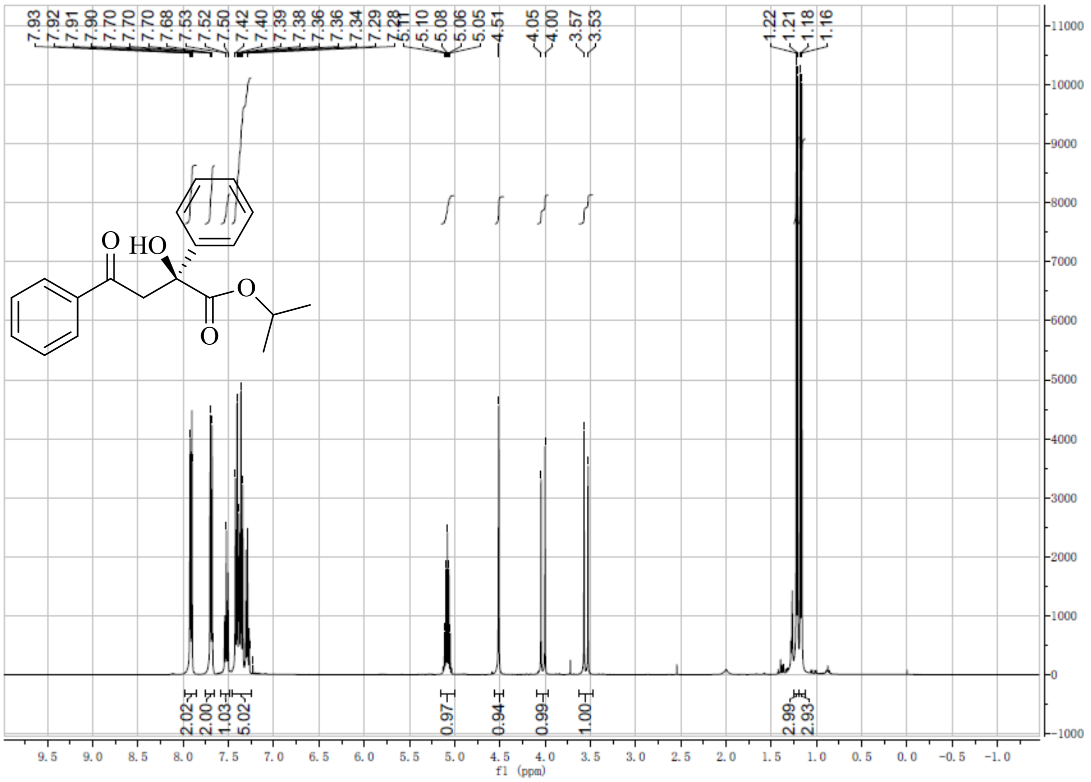

^{13}C NMR of **3p**

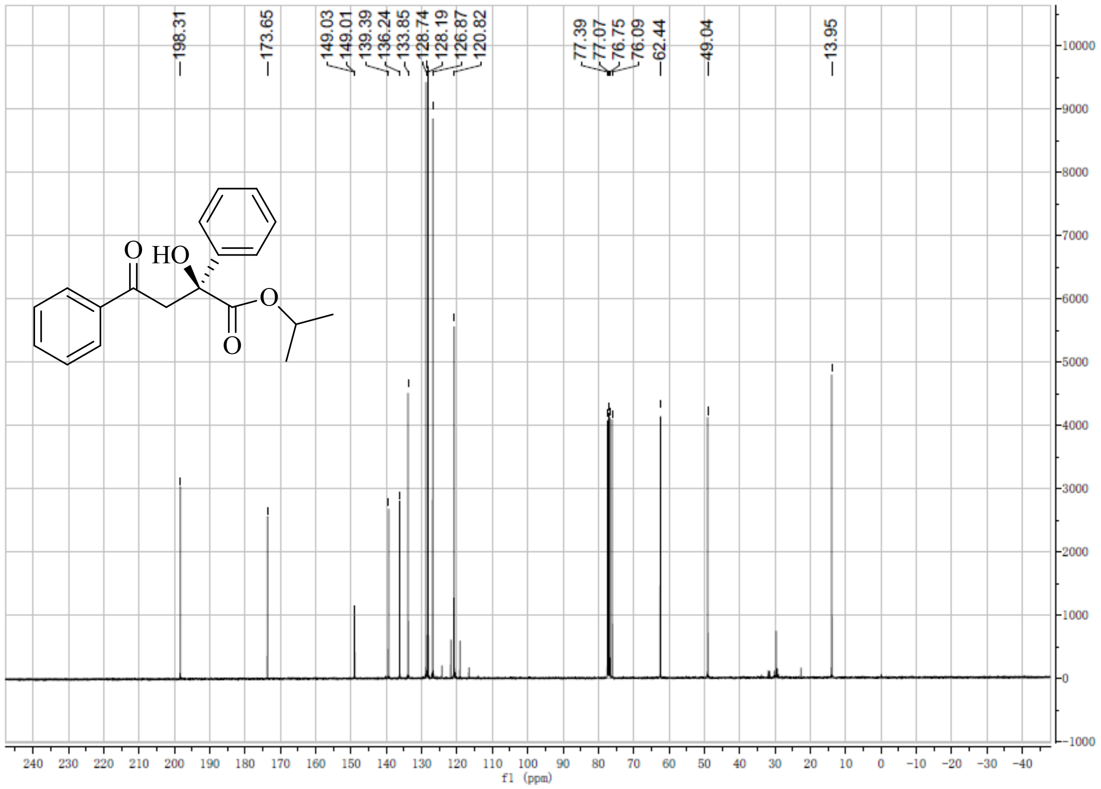

^{1}H NMR of **3q**

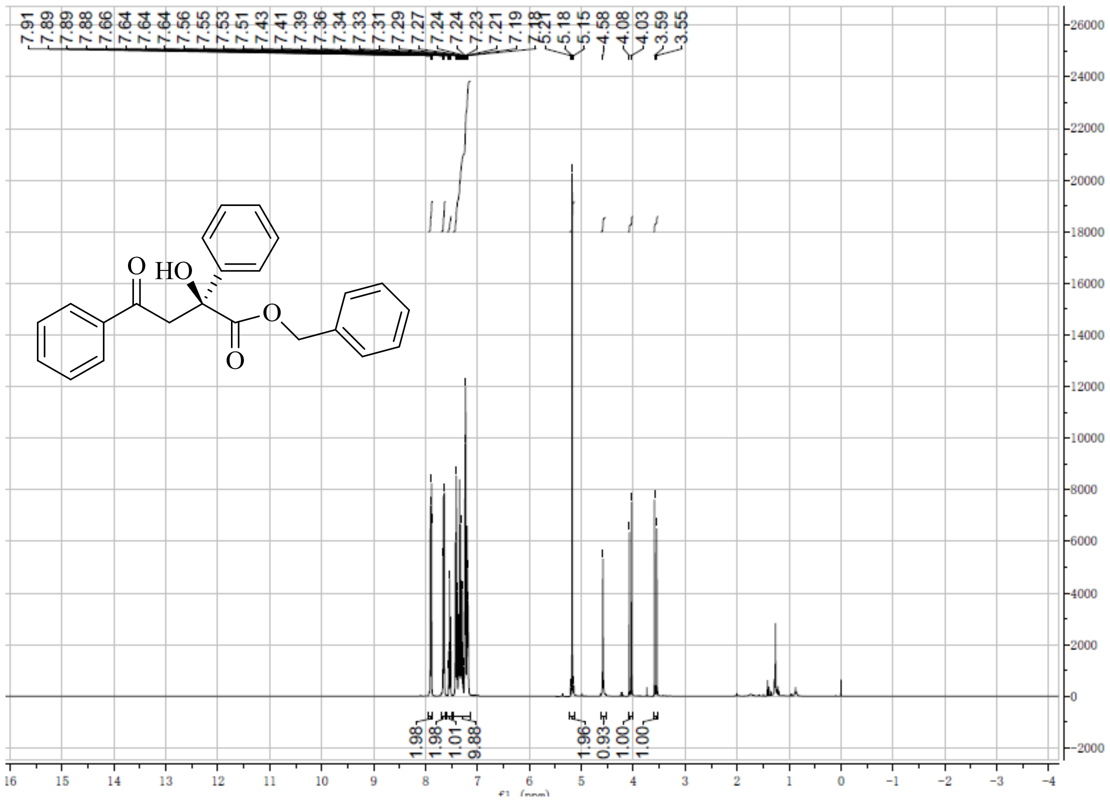

^{13}C NMR of **3q**

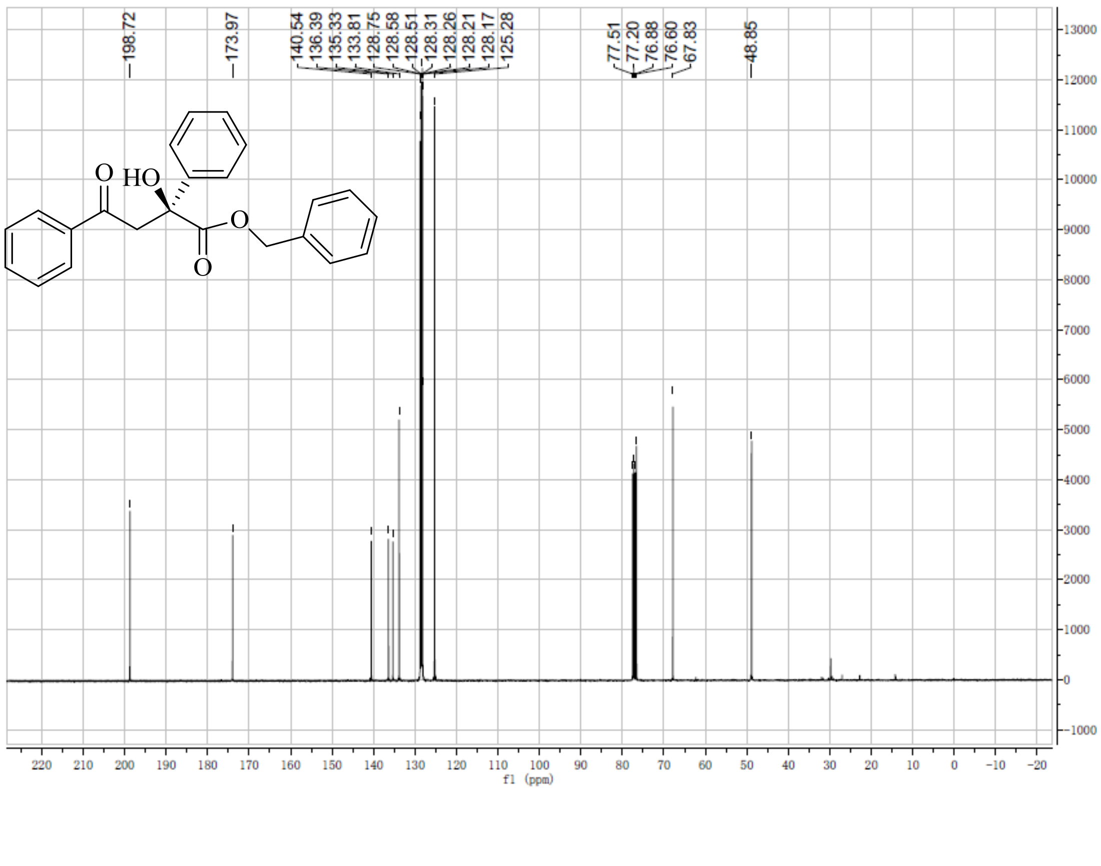

4. HPLC of compound 3

HPLC of compound 3a

<Chromatogram>

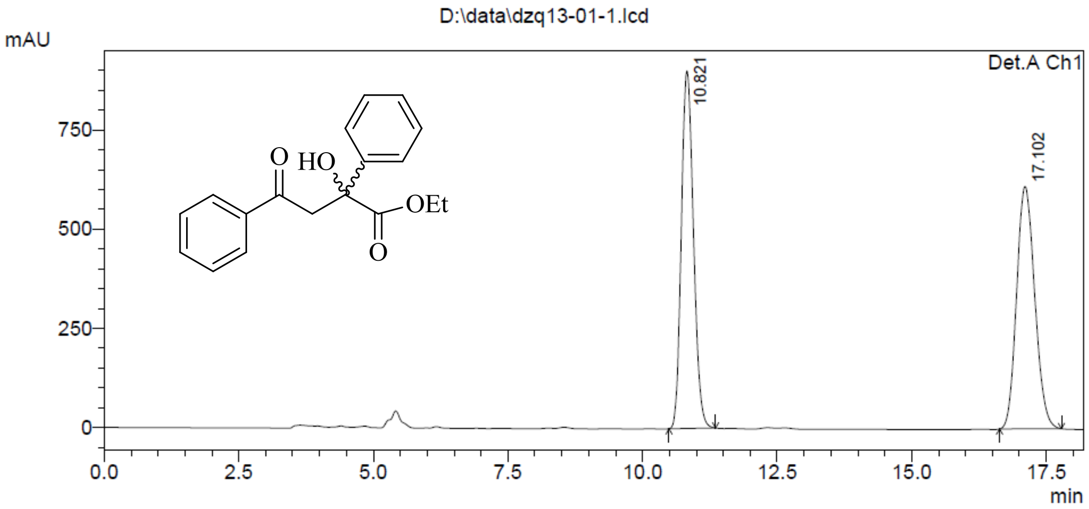

1 Det.A Ch1/254nm

PeakTable

Detector A Ch1 254nm

Peak#	Ret. Time	Area	Height	Area %	Height %
1	10.821	14128002	901987	49.628	59.607
2	17.102	14339641	611231	50.372	40.393
Total		28467643	1513218	100.000	100.000

<Chromatogram>

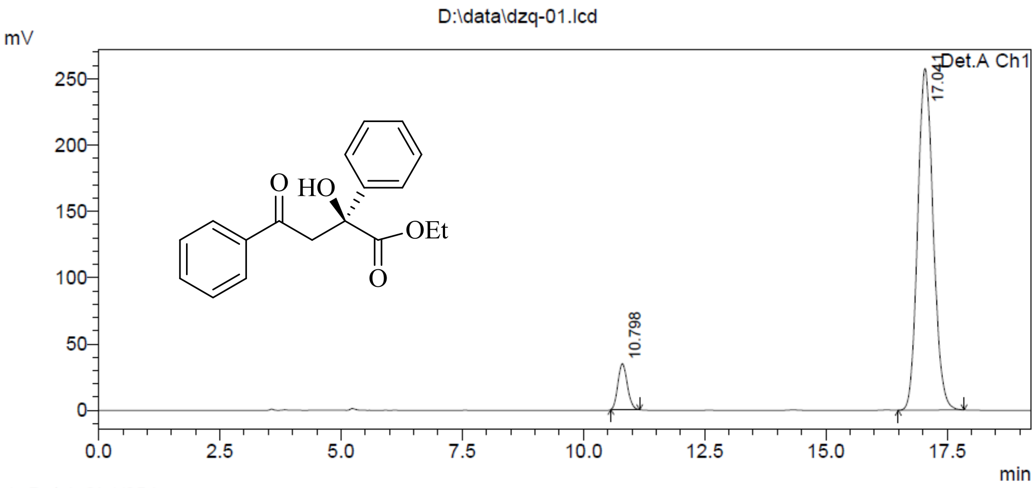

1 Det.A Ch1/254nm

PeakTable

Detector A Ch1 254nm

Peak#	Ret. Time	Area	Height	Area %	Height %
1	10.798	468898	34622	7.547	11.851
2	17.041	5743907	257517	92.453	88.149
Total		6212805	292139	100.000	100.000

HPLC of compound 3b

<Chromatogram>

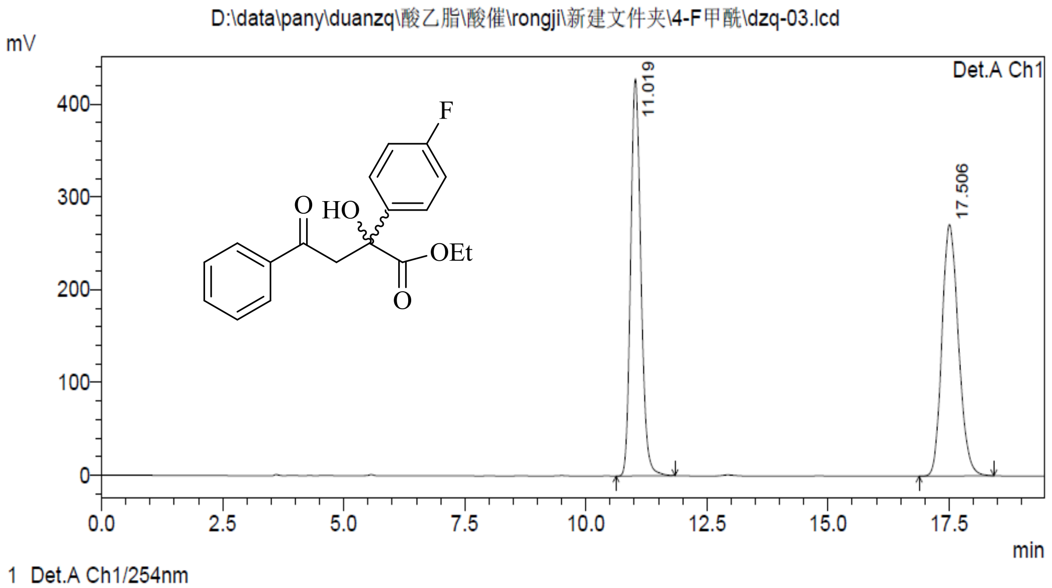

1 Det.A Ch1/254nm

PeakTable

Detector A Ch1 254nm

Peak#	Ret. Time	Area	Height	Area %	Height %
1	11.019	6326467	428494	49.600	61.235
2	17.506	6428572	271264	50.400	38.765
Total		12755039	699758	100.000	100.000

<Chromatogram>

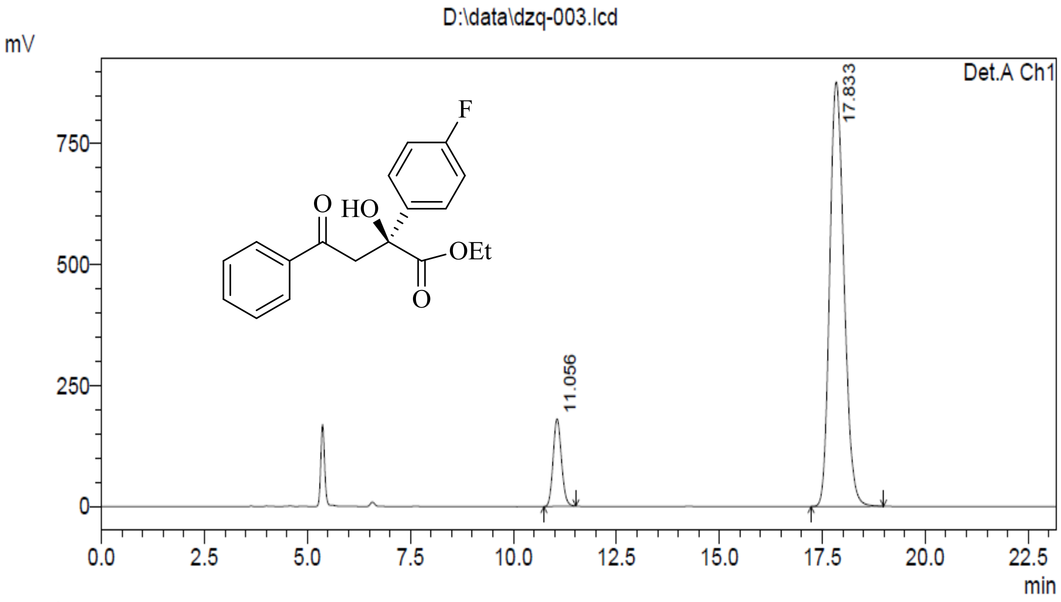

1 Det.A Ch1/254nm

PeakTable

Detector A Ch1 254nm

Peak#	Ret. Time	Area	Height	Area %	Height %
1	11.056	2550054	180621	10.724	17.073
2	17.833	21229374	877310	89.276	82.927
Total		23779428	1057932	100.000	100.000

HPLC of compound 3c

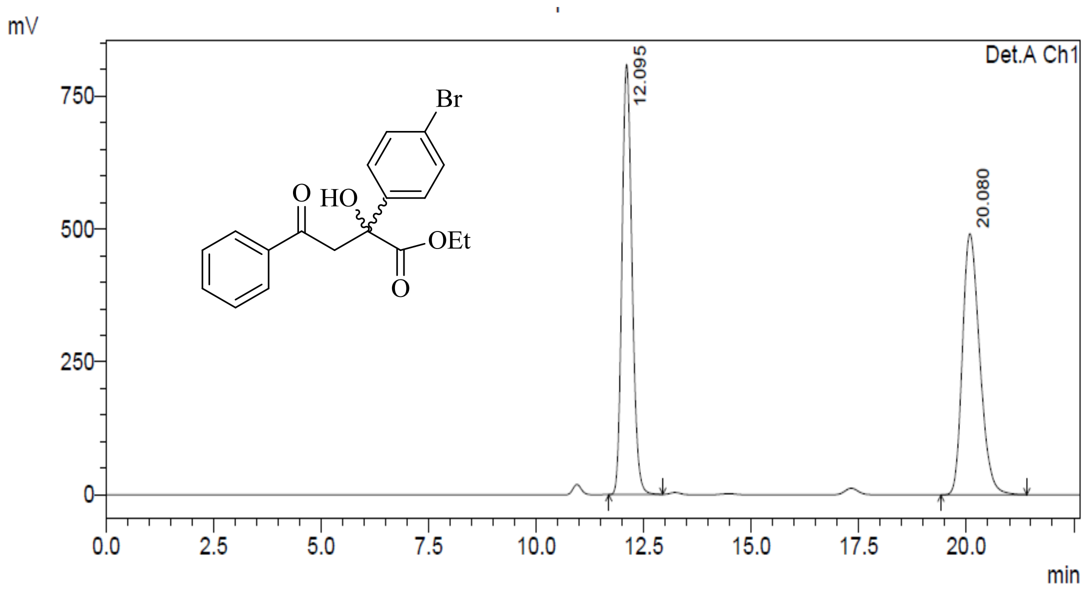

1 Det.A Ch1/254nm

PeakTable

Detector A Ch1 254nm

Peak#	Ret. Time	Area	Height	Area %	Height %
1	12.095	13181962	809066	49.039	62.227
2	20.080	13698358	491128	50.961	37.773
Total		26880319	1300194	100.000	100.000

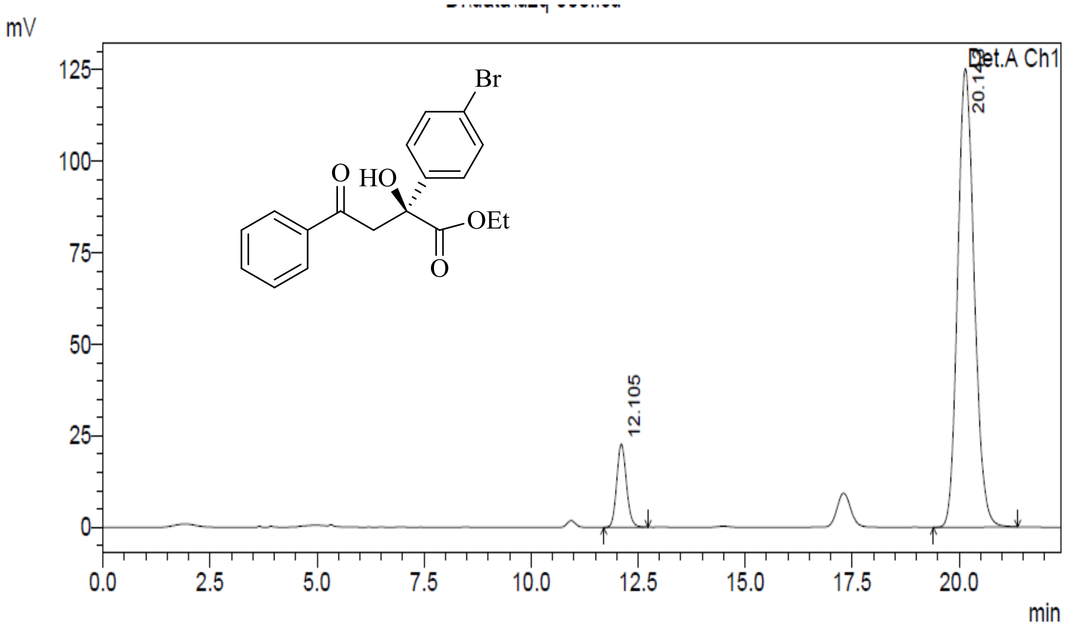

1 Det.A Ch1/254nm

PeakTable

Detector A Ch1 254nm

Peak#	Ret. Time	Area	Height	Area %	Height %
1	12.105	360076	22764	9.623	15.367
2	20.143	3381561	125377	90.377	84.633
Total		3741638	148142	100.000	100.000

HPLC of compound 3d

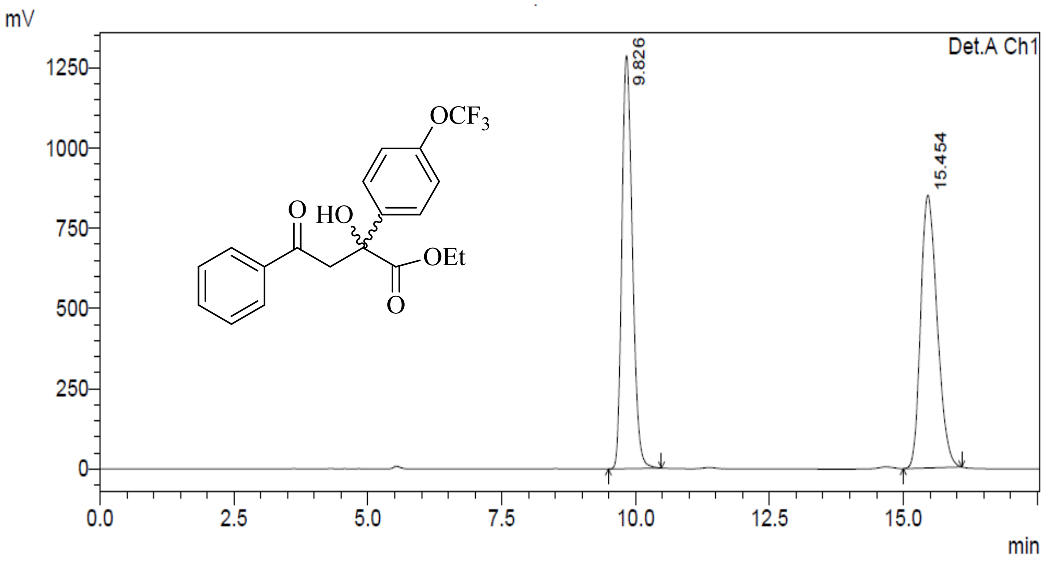

1 Det.A Ch1/254nm

PeakTable

Detector A Ch1 254nm

Peak#	Ret. Time	Area	Height	Area %	Height %
1	9.826	17589340	1286256	49.128	60.221
2	15.454	18213385	849631	50.872	39.779
Total		35802725	2135887	100.000	100.000

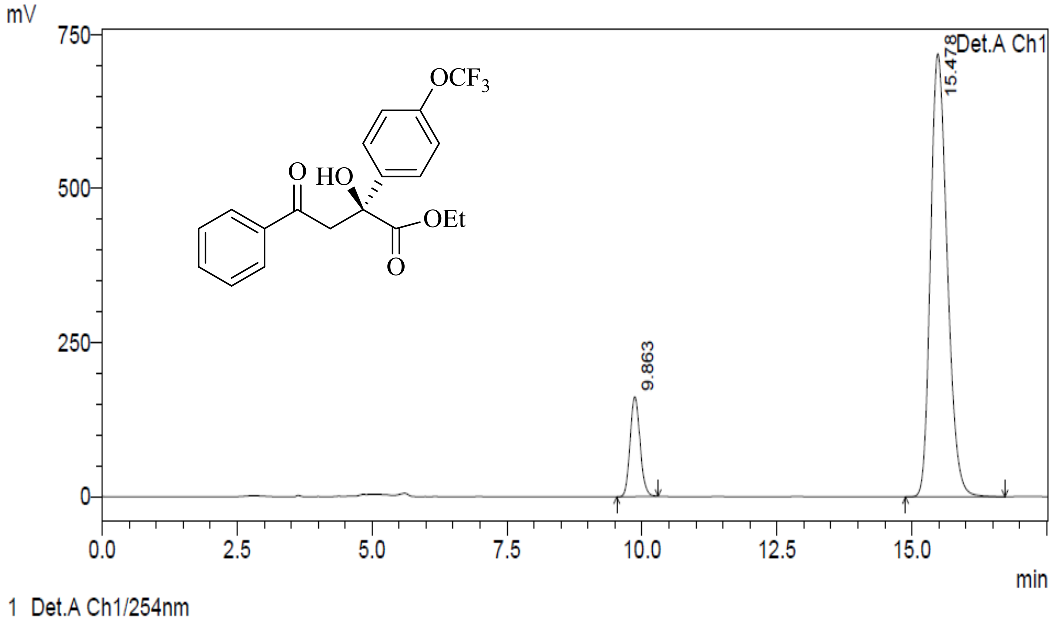

1 Det.A Ch1/254nm

PeakTable

Detector A Ch1 254nm

Peak#	Ret. Time	Area	Height	Area %	Height %
1	9.863	2087854	162163	11.875	18.395
2	15.478	15494751	719408	88.125	81.605
Total		17582605	881571	100.000	100.000

HPLC of compound 3e

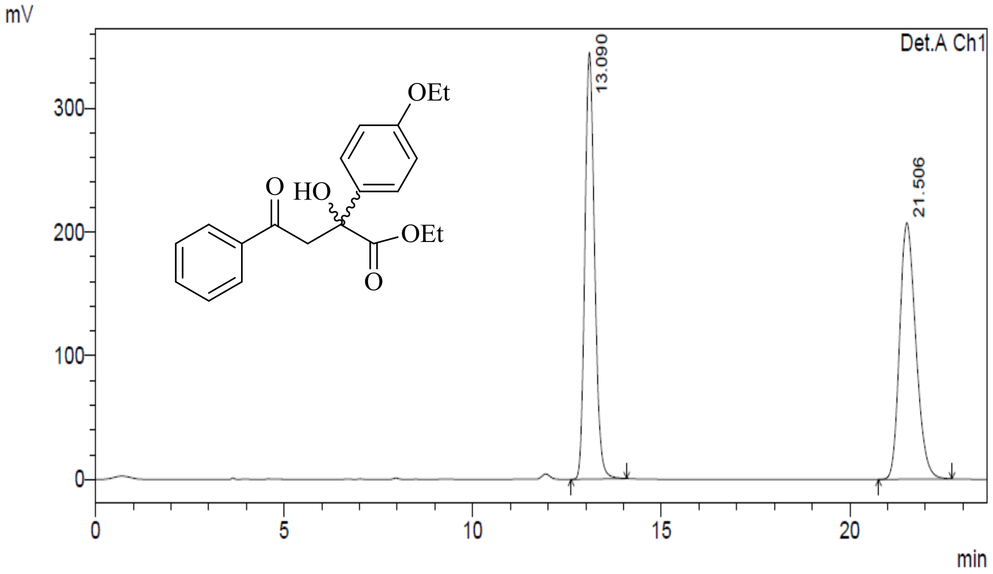

1 Det.A Ch1/254nm

PeakTable

Detector A Ch1 254nm

Peak#	Ret. Time	Area	Height	Area %	Height %
1	13.090	6154822	344752	49.818	62.448
2	21.506	6199699	207309	50.182	37.552
Total		12354521	552061	100.000	100.000

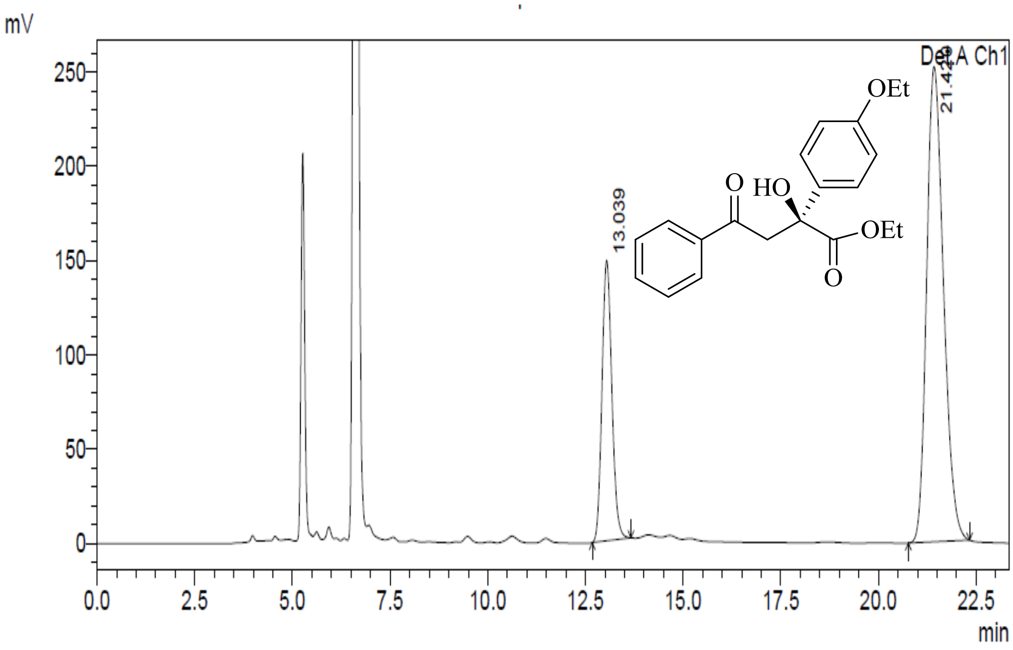

1 Det.A Ch1/254nm

PeakTable

Detector A Ch1 254nm

Peak#	Ret. Time	Area	Height	Area %	Height %
1	13.039	2604314	148664	25.646	37.116
2	21.420	7550398	251871	74.354	62.884
Total		10154712	400535	100.000	100.000

HPLC of compound 3f

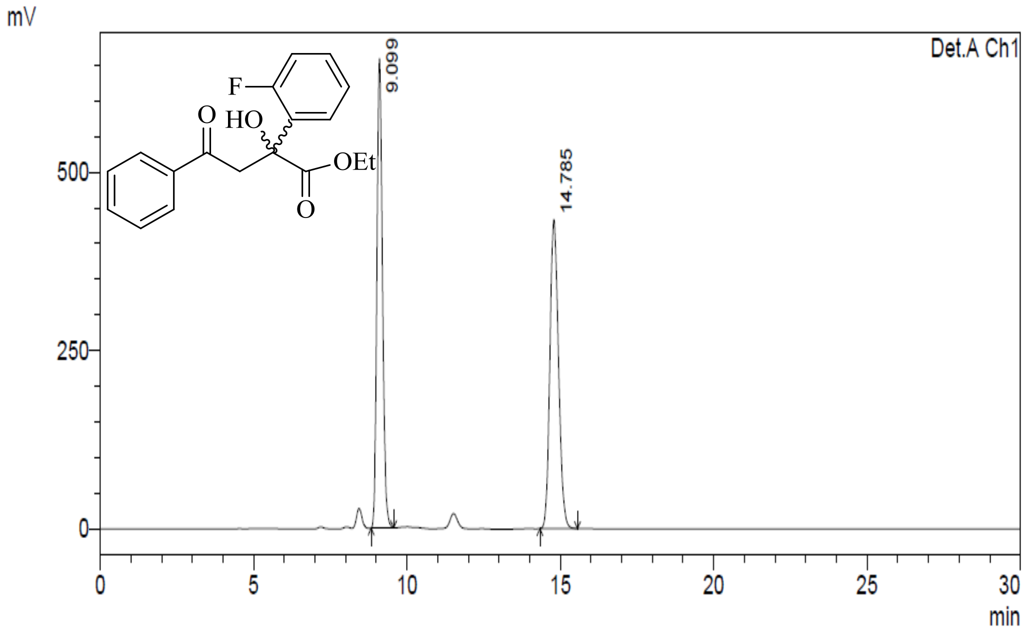

Peak#	Ret. Time	Area	Height	Area %	Height %
1	9.099	8316163	657847	49.536	60.291
2	14.785	8472016	433271	50.464	39.709
Total		16788178	1091118	100.000	100.000

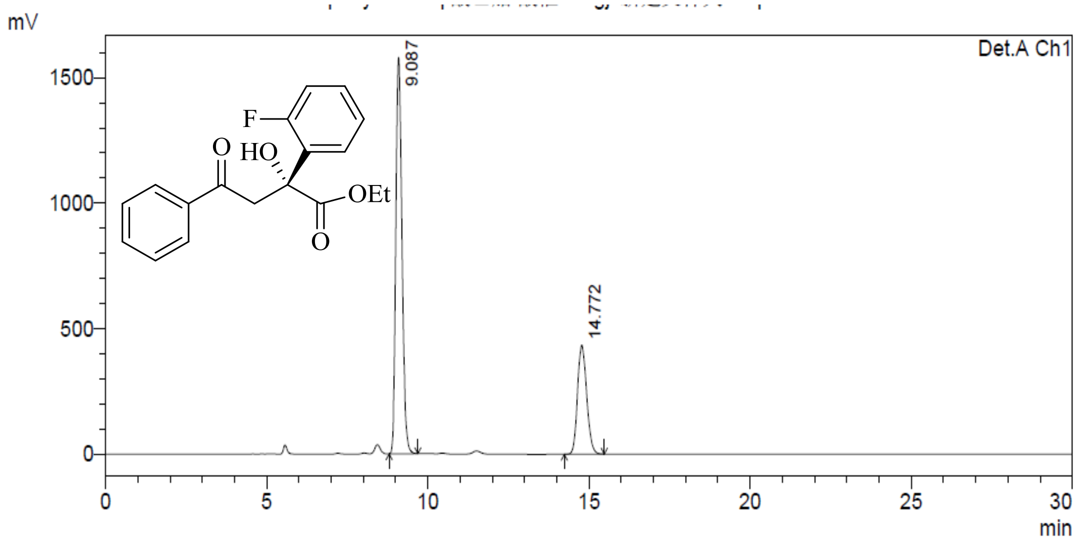

Peak#	Ret. Time	Area	Height	Area %	Height %
1	9.087	21003486	1579061	70.934	78.409
2	14.772	8606317	434815	29.066	21.591
Total		29609803	2013876	100.000	100.000

HPLC of compound 3g

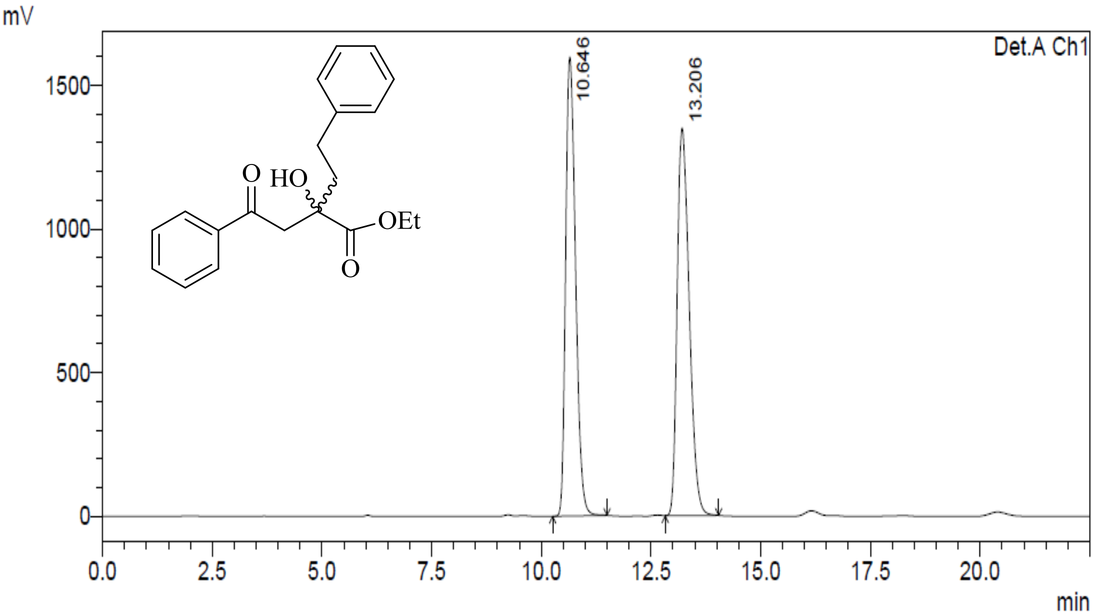

1 Det.A Ch1/254nm

PeakTable

Detector A Ch1 254nm

Peak#	Ret. Time	Area	Height	Area %	Height %
1	10.646	25078969	1597216	49.252	54.230
2	13.206	25840979	1348025	50.748	45.770
Total		50919948	2945241	100.000	100.000

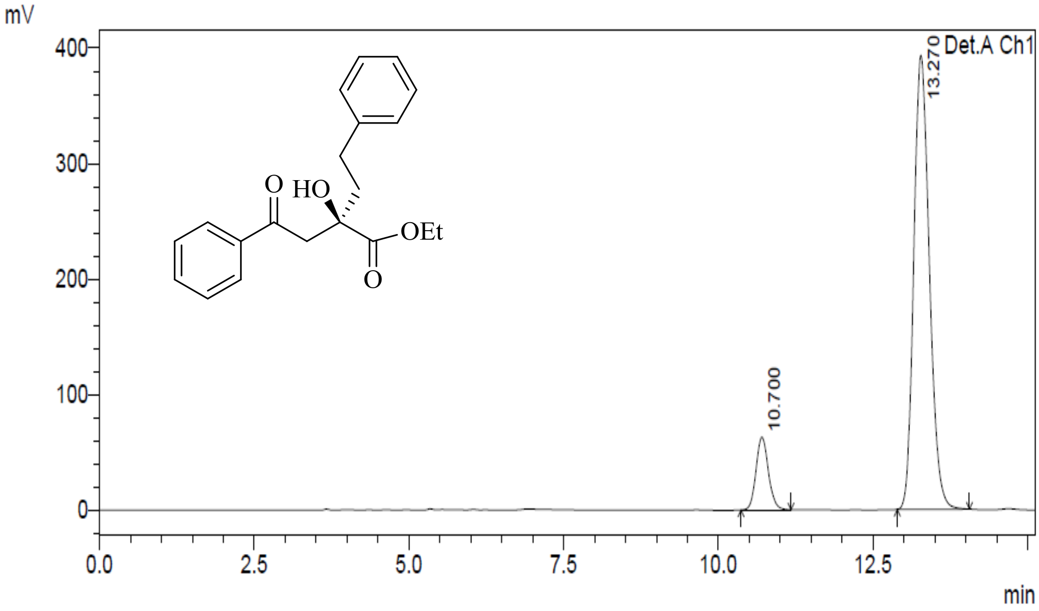

1 Det.A Ch1/254nm

PeakTable

Detector A Ch1 254nm

Peak#	Ret. Time	Area	Height	Area %	Height %
1	10.700	877709	62934	11.295	13.803
2	13.270	6893196	393001	88.705	86.197
Total		7770905	455935	100.000	100.000

HPLC of compound 3h

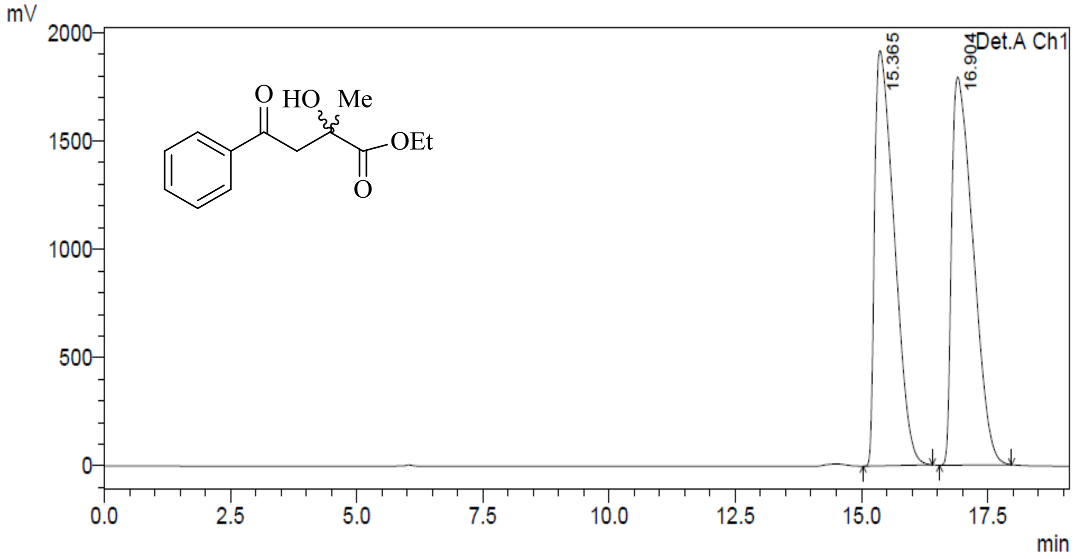

1 Det.A Ch1/254nm

PeakTable

Detector A Ch1 254nm

Peak#	Ret. Time	Area	Height	Area %	Height %
1	15.365	51886679	1917324	49.400	51.683
2	16.904	53146470	1792446	50.600	48.317
Total		105033149	3709770	100.000	100.000

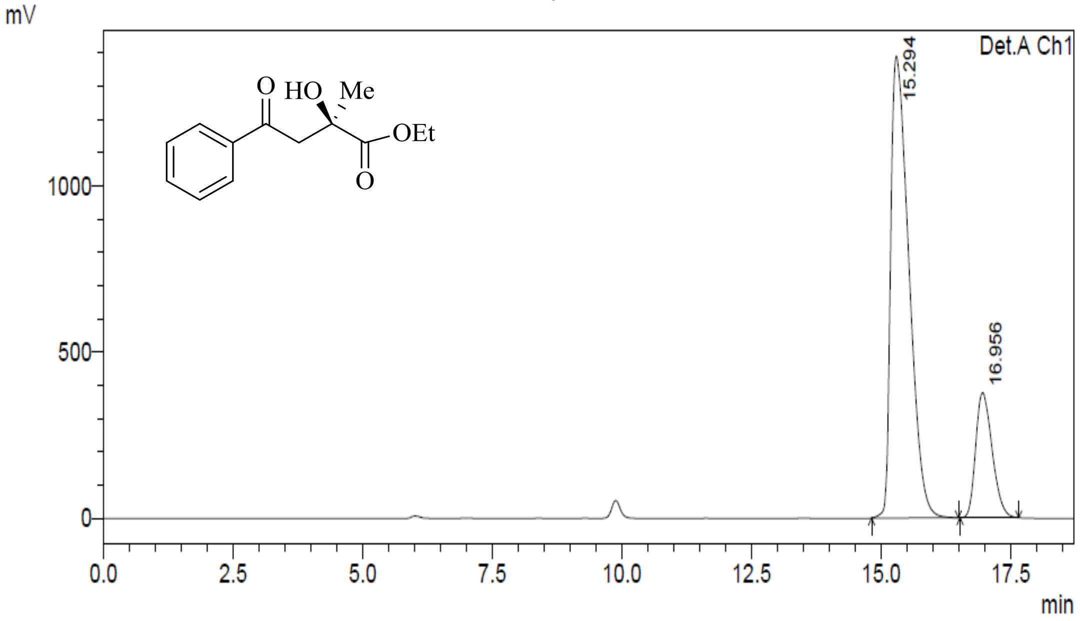

1 Det.A Ch1/254nm

PeakTable

Detector A Ch1 254nm

Peak#	Ret. Time	Area	Height	Area %	Height %
1	15.294	33984800	1389958	80.272	78.682
2	16.956	8352470	376584	19.728	21.318
Total		42337270	1766542	100.000	100.000

HPLC of compound 3i

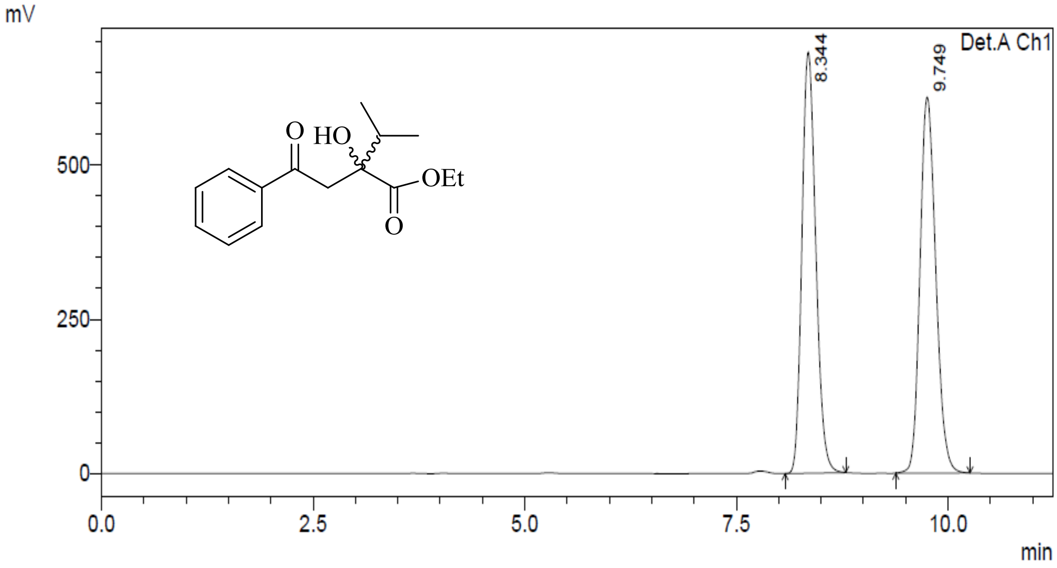

Peak#	Ret. Time	Area	Height	Area %	Height %
1	8.344	7754989	683015	49.514	52.850
2	9.749	7907074	609355	50.486	47.150
Total		15662063	1292370	100.000	100.000

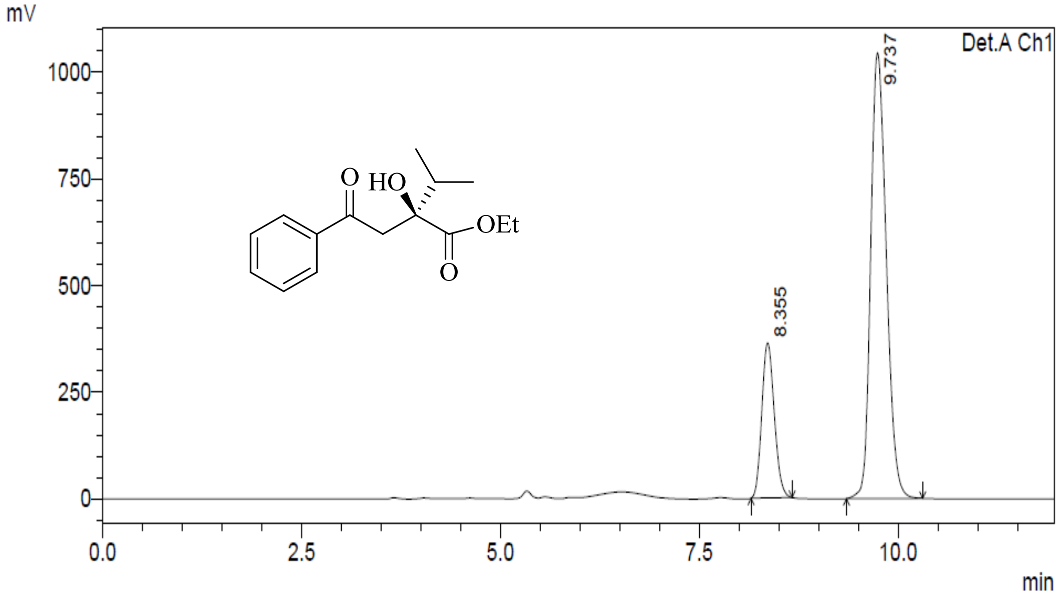

Peak#	Ret. Time	Area	Height	Area %	Height %
1	8.355	3972905	363191	21.780	25.799
2	9.737	14268331	1044586	78.220	74.201
Total		18241235	1407777	100.000	100.000

HPLC of compound 3j

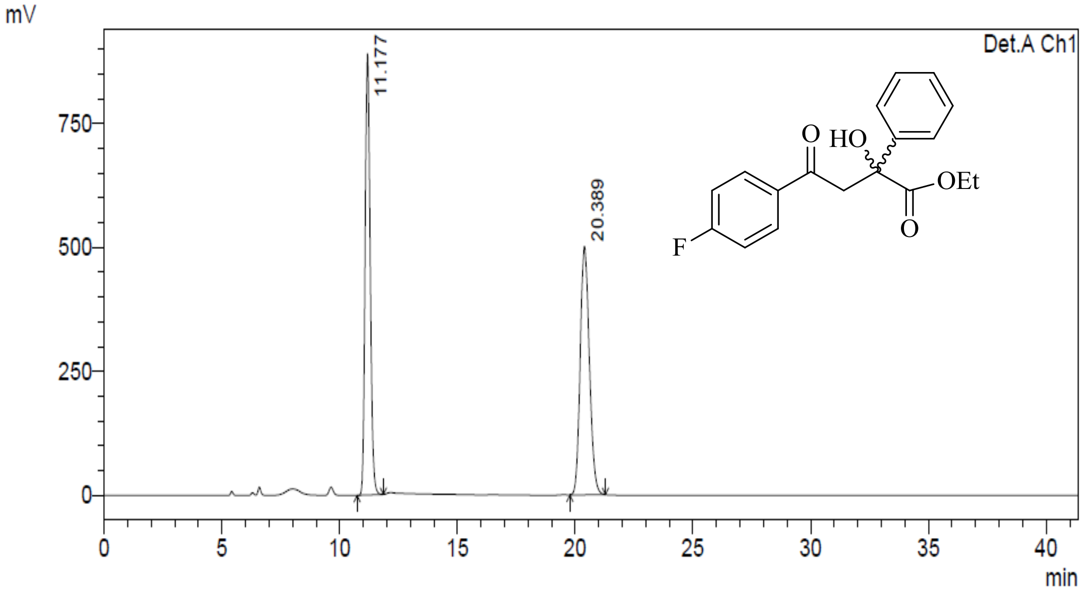

1 Det.A Ch1/254nm

PeakTable

Detector A Ch1 254nm

Peak#	Ret. Time	Area	Height	Area %	Height %
1	11.177	13423708	889830	49.692	63.986
2	20.389	13590263	500837	50.308	36.014
Total		27013971	1390667	100.000	100.000

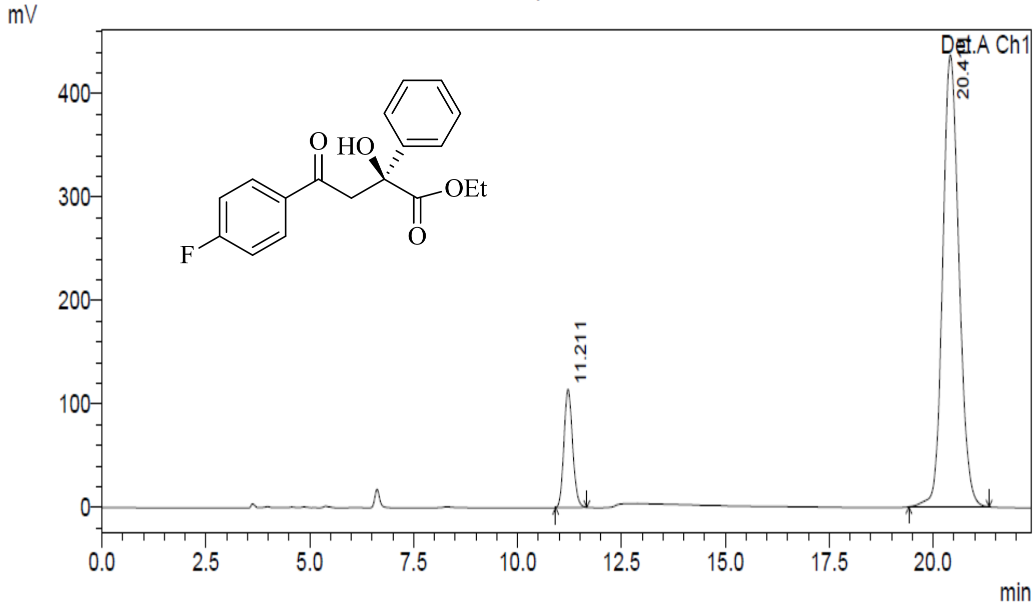

1 Det.A Ch1/254nm

PeakTable

Detector A Ch1 254nm

Peak#	Ret. Time	Area	Height	Area %	Height %
1	11.211	1661930	114353	12.245	20.751
2	20.411	11910520	436718	87.755	79.249
Total		13572450	551071	100.000	100.000

HPLC of compound 3k

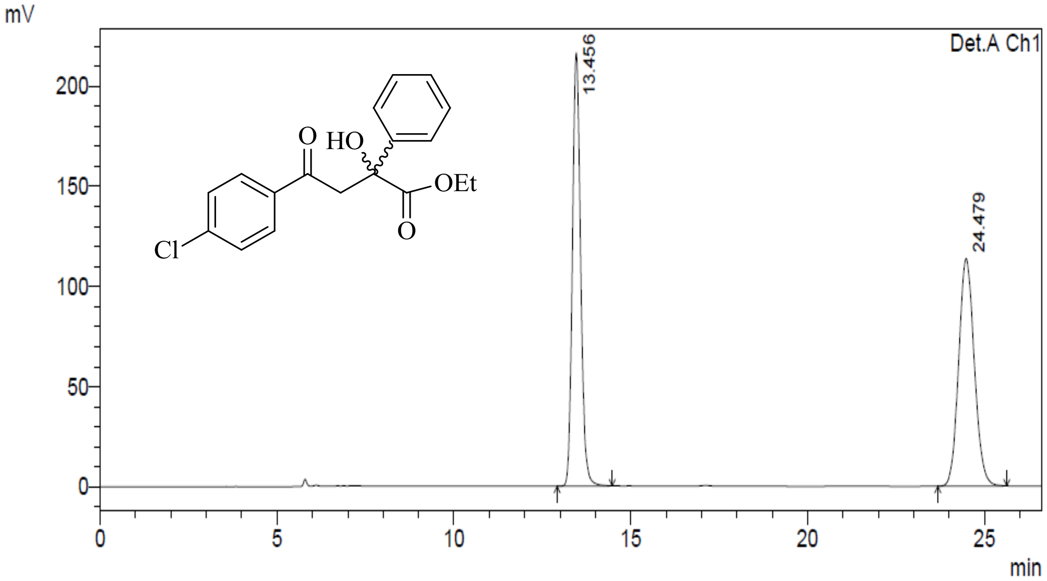

1 Det.A Ch1/254nm

PeakTable

Detector A Ch1 254nm

Peak#	Ret. Time	Area	Height	Area %	Height %
1	13.456	3493508	216226	49.966	65.565
2	24.479	3498250	113563	50.034	34.435
Total		6991758	329789	100.000	100.000

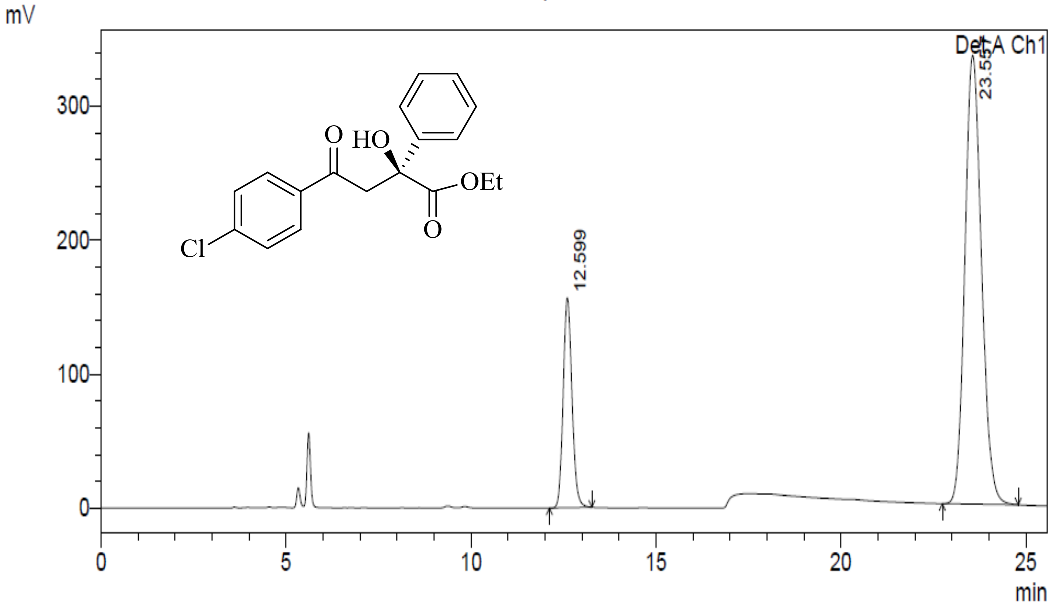

1 Det.A Ch1/254nm

PeakTable

Detector A Ch1 254nm

Peak#	Ret. Time	Area	Height	Area %	Height %
1	12.599	2604384	156569	19.918	31.855
2	23.557	10471455	334931	80.082	68.145
Total		13075839	491500	100.000	100.000

HPLC of compound 3l

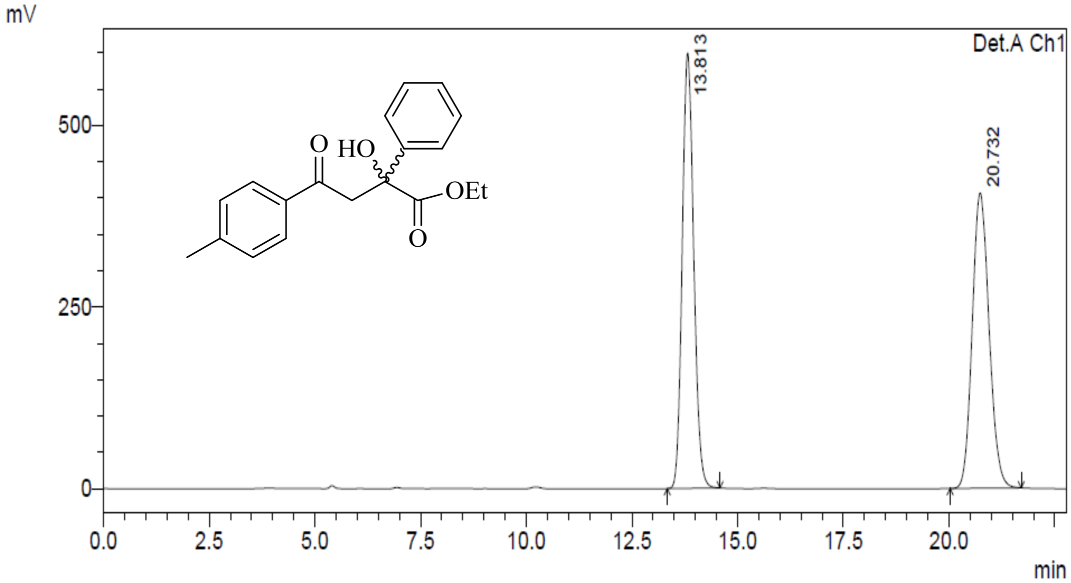

1 Det.A Ch1/254nm

PeakTable

Detector A Ch1 254nm

Peak#	Ret. Time	Area	Height	Area %	Height %
1	13.813	11317806	599291	50.075	59.588
2	20.732	11283893	406429	49.925	40.412
Total		22601698	1005720	100.000	100.000

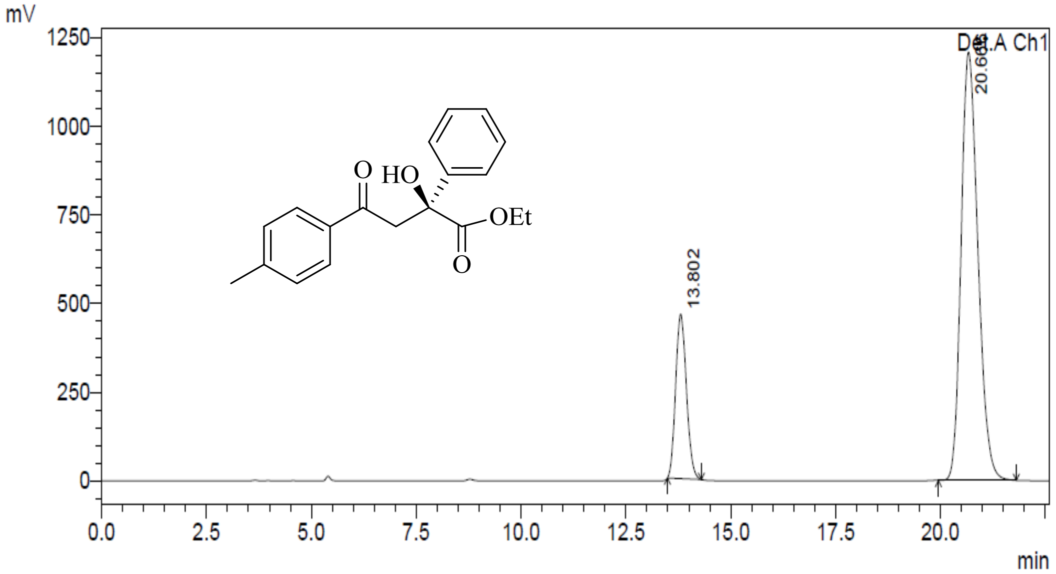

1 Det.A Ch1/254nm

PeakTable

Detector A Ch1 254nm

Peak#	Ret. Time	Area	Height	Area %	Height %
1	13.802	8314498	463588	19.782	27.748
2	20.666	33716936	1207103	80.218	72.252
Total		42031435	1670692	100.000	100.000

HPLC of compound 3m

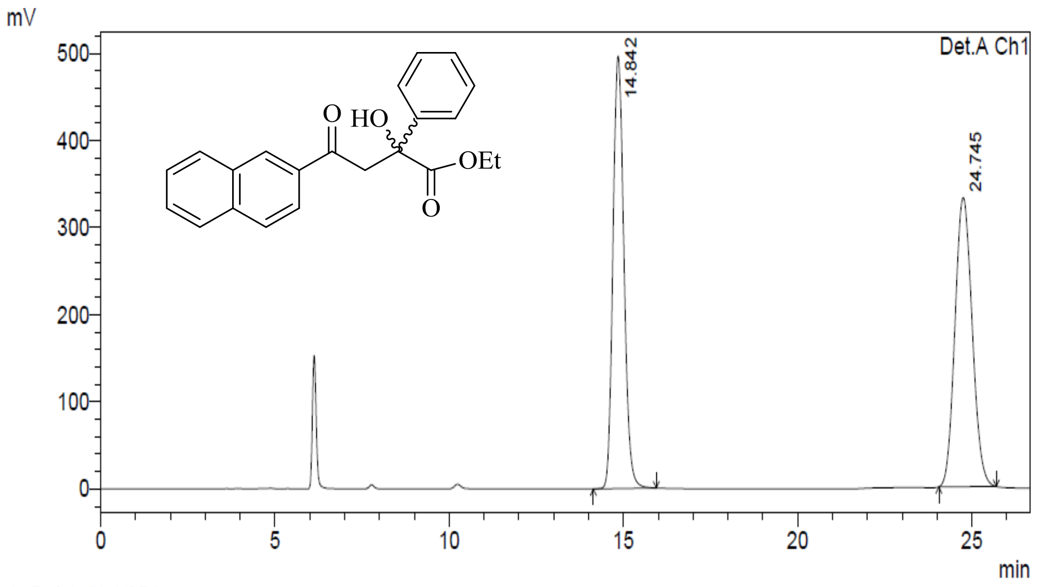

1 Det.A Ch1/254nm

PeakTable

Detector A Ch1 254nm

Peak#	Ret. Time	Area	Height	Area %	Height %
1	14.842	10845763	496444	48.682	59.933
2	24.745	11432865	331891	51.318	40.067
Total		22278628	828335	100.000	100.000

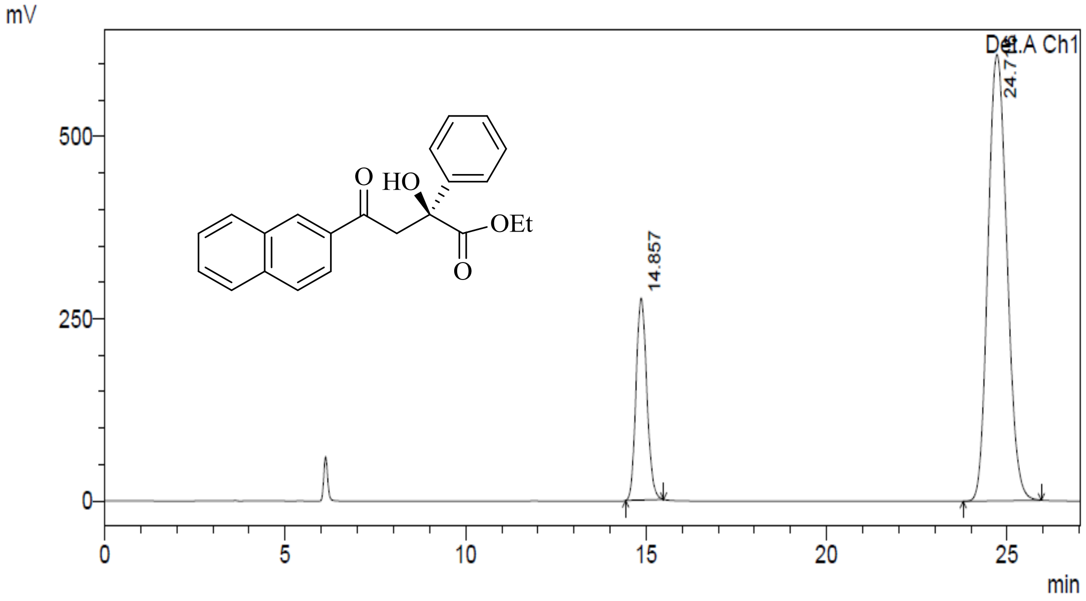

1 Det.A Ch1/254nm

PeakTable

Detector A Ch1 254nm

Peak#	Ret. Time	Area	Height	Area %	Height %
1	14.857	5782639	277289	20.518	31.160
2	24.716	22400746	612585	79.482	68.840
Total		28183385	889874	100.000	100.000

HPLC of compound 3m

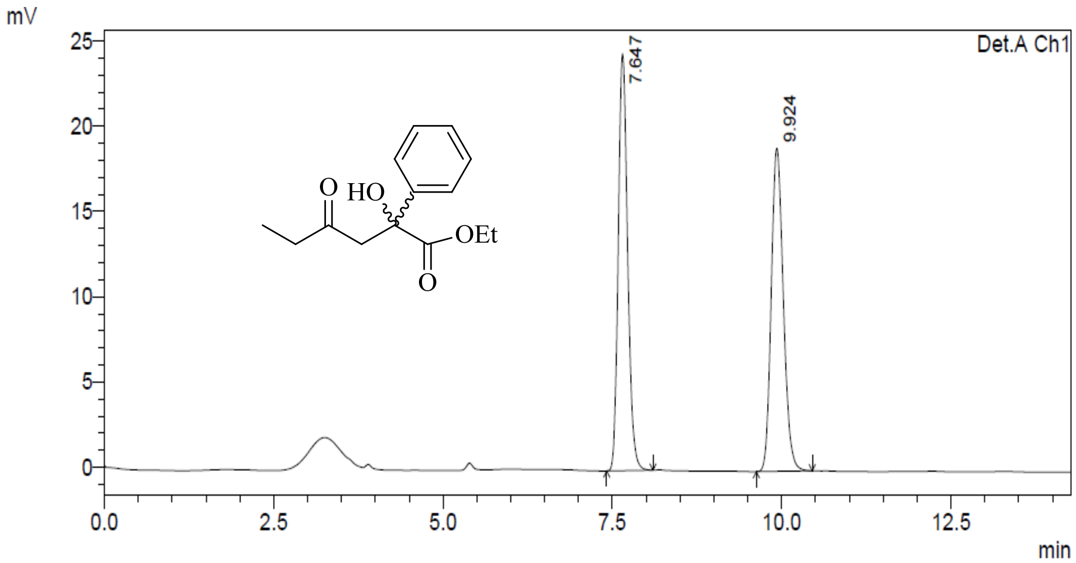

1 Det.A Ch1/254nm

PeakTable

Detector A Ch1 254nm

Peak#	Ret. Time	Area	Height	Area %	Height %
1	7.647	235284	24476	49.985	56.365
2	9.924	235425	18949	50.015	43.635
Total		470709	43425	100.000	100.000

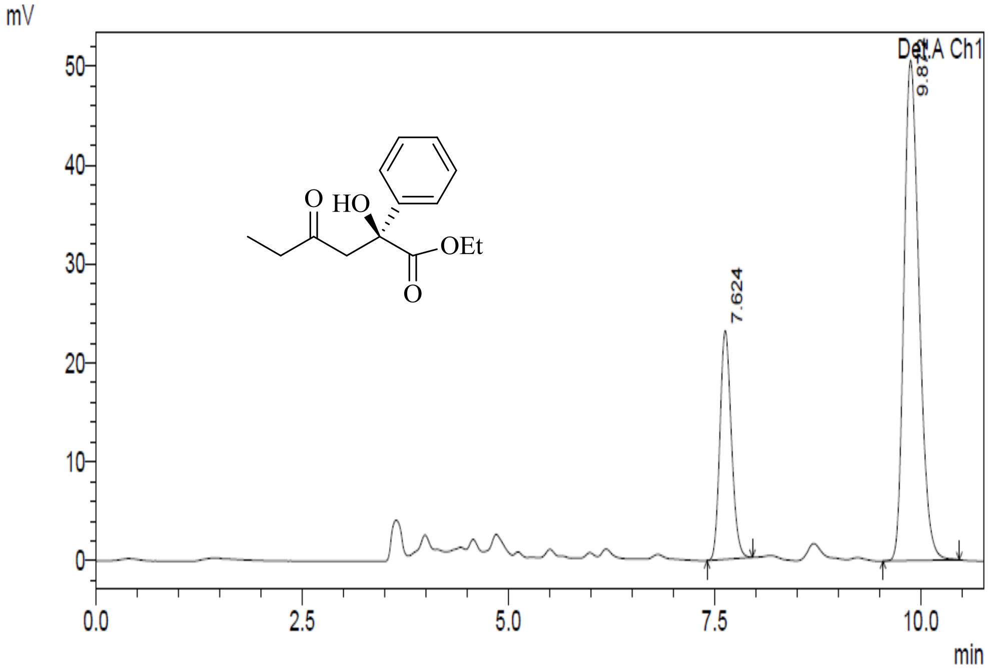

1 Det.A Ch1/254nm

PeakTable

Detector A Ch1 254nm

Peak#	Ret. Time	Area	Height	Area %	Height %
1	7.624	223534	23122	25.682	31.368
2	9.872	646873	50591	74.318	68.632
Total		870407	73714	100.000	100.000

HPLC of compound 3o

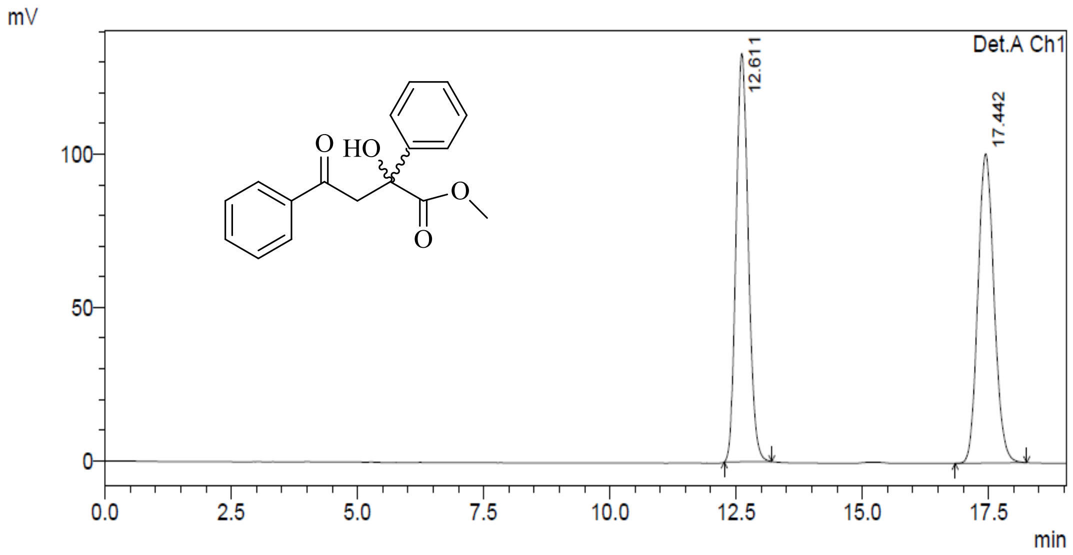

Peak#	Ret. Time	Area	Height	Area %	Height %
1	12.611	2248377	133194	49.955	56.917
2	17.442	2252436	100819	50.045	43.083
Total		4500813	234013	100.000	100.000

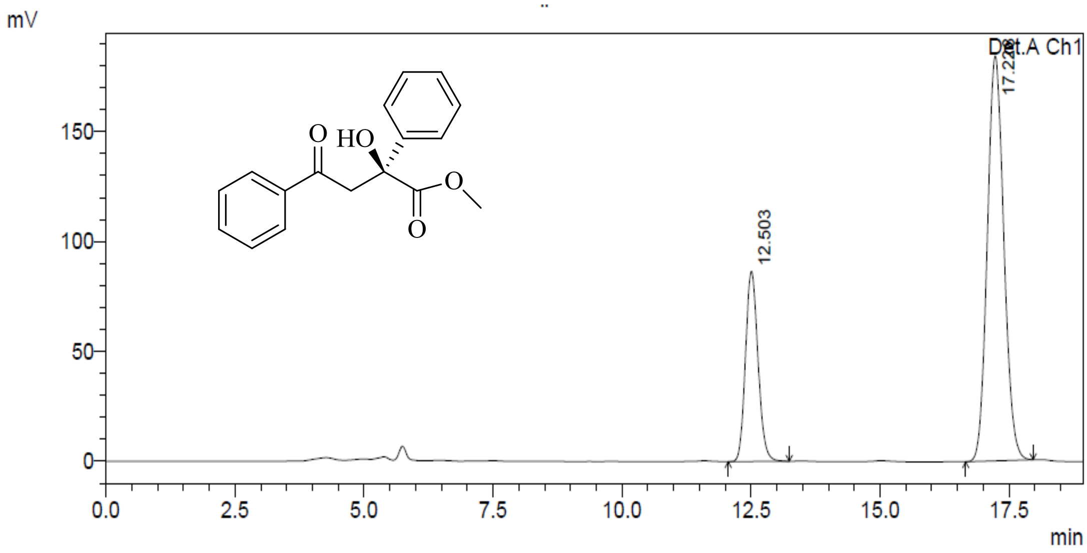

Peak#	Ret. Time	Area	Height	Area %	Height %
1	12.503	1463410	86553	26.248	31.972
2	17.228	4111848	184164	73.752	68.028
Total		5575258	270717	100.000	100.000

HPLC of compound 3p

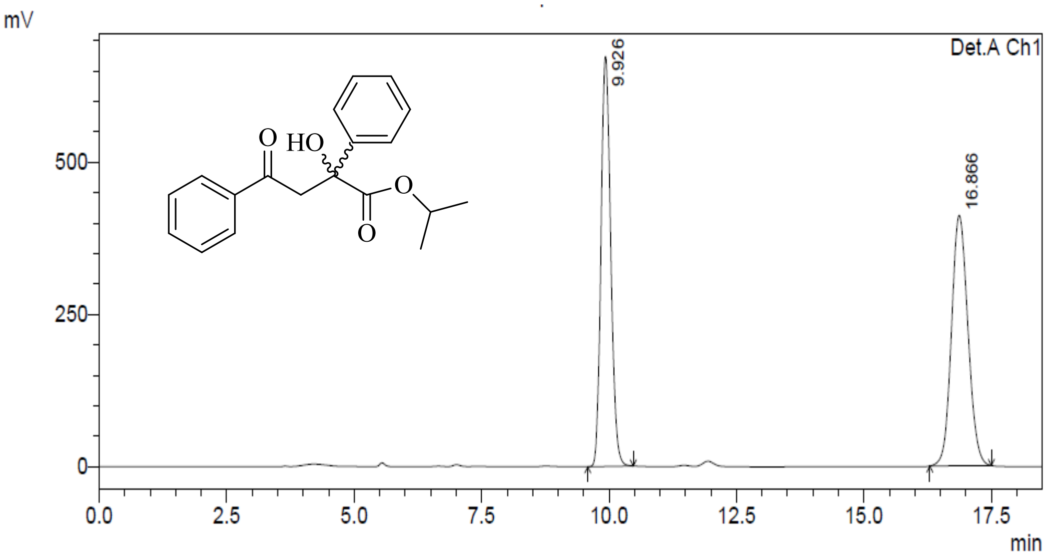

1 Det.A Ch1/254nm

PeakTable

Detector A Ch1 254nm

Peak#	Ret. Time	Area	Height	Area %	Height %
1	9.926	8938758	673743	49.144	62.065
2	16.866	9250017	411805	50.856	37.935
Total		18188775	1085547	100.000	100.000

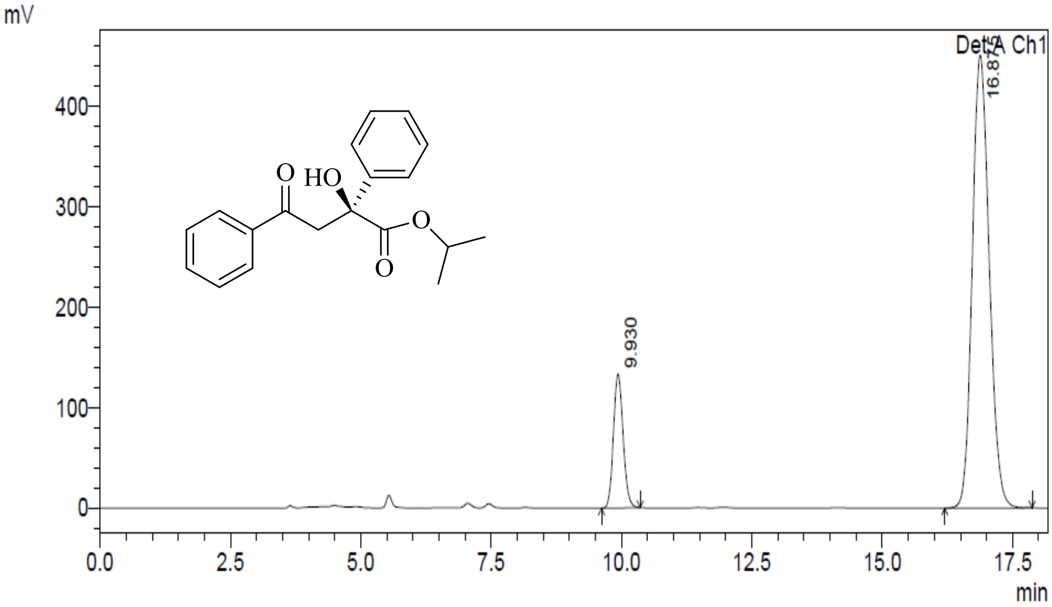

1 Det.A Ch1/254nm

PeakTable

Detector A Ch1 254nm

Peak#	Ret. Time	Area	Height	Area %	Height %
1	9.930	1723813	133501	14.457	22.837
2	16.875	10199878	451084	85.543	77.163
Total		11923691	584584	100.000	100.000

HPLC of compound 3q

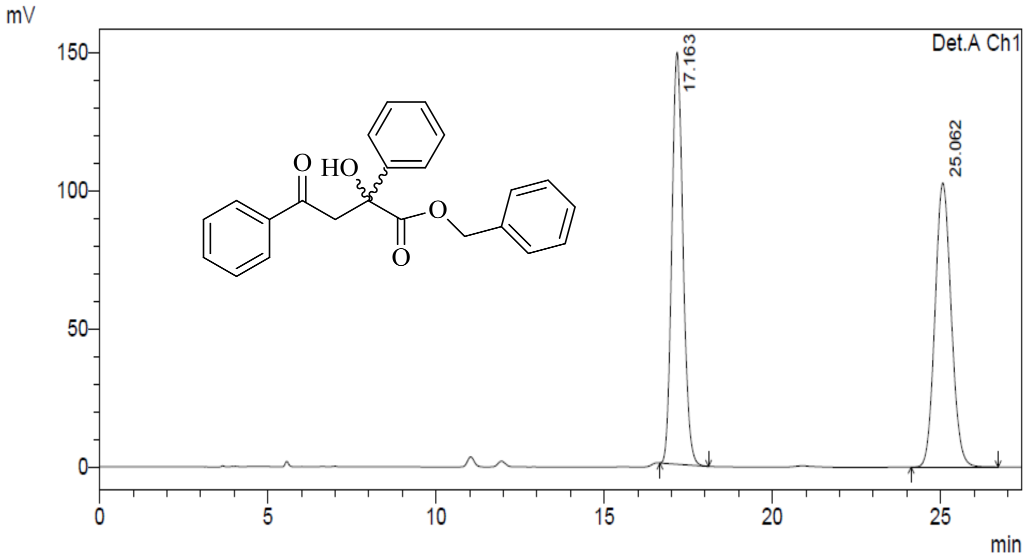

1 Det.A Ch1/254nm

PeakTable

Detector A Ch1 254nm

Peak#	Ret. Time	Area	Height	Area %	Height %
1	17.163	3431995	149056	49.903	59.178
2	25.062	3445339	102822	50.097	40.822
Total		6877334	251878	100.000	100.000

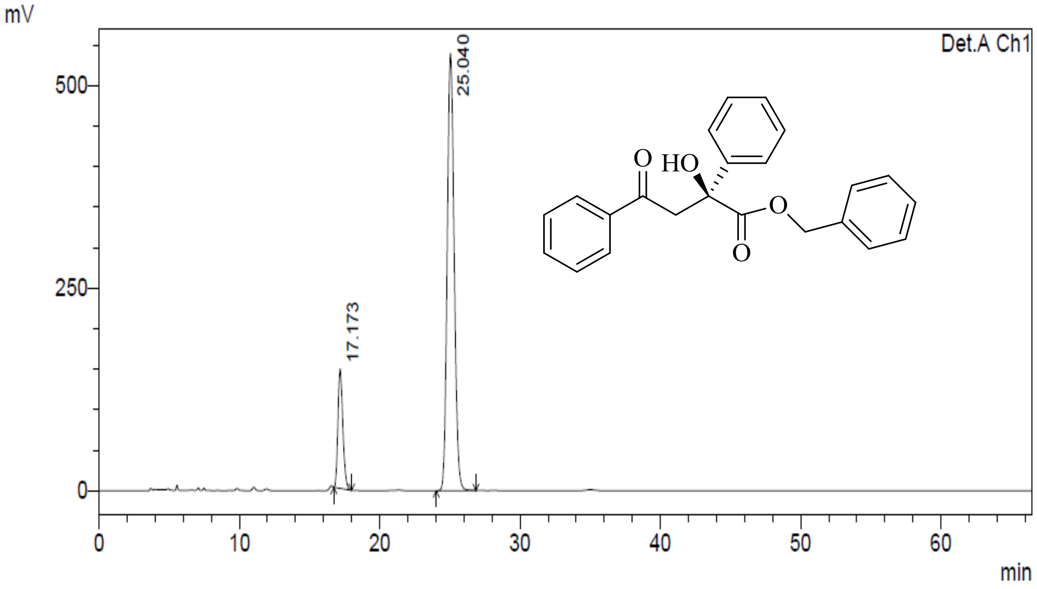

1 Det.A Ch1/254nm

PeakTable

Detector A Ch1 254nm

Peak#	Ret. Time	Area	Height	Area %	Height %
1	17.173	3583779	147483	16.158	21.448
2	25.040	18595143	540136	83.842	78.552
Total		22178922	687619	100.000	100.000
